# Understanding Community Norms Surrounding Tobacco Sales

**DOI:** 10.1371/journal.pone.0106461

**Published:** 2014-09-02

**Authors:** Patricia A. McDaniel, Ruth E. Malone

**Affiliations:** Department of Social and Behavioral Sciences, School of Nursing, University of California San Francisco, San Francisco, California, United States of America; Medical University of Vienna, Austria

## Abstract

**Background:**

In the US, denormalizing tobacco use is key to tobacco control; less attention has been paid to denormalizing tobacco sales. However, some localities have placed limits on the number and type of retailers who may sell tobacco, and some retailers have abandoned tobacco sales voluntarily. Understanding community norms surrounding tobacco sales may help accelerate tobacco denormalization.

**Methods:**

We conducted 15 focus groups with customers of California, New York, and Ohio retailers who had voluntarily discontinued tobacco sales to examine normative assumptions about where cigarettes should or should not be sold, voluntary decisions to discontinue tobacco sales, and government limits on such sales.

**Results:**

Groups in all three states generally agreed that grocery stores that sold healthy products should not sell tobacco; California groups saw pharmacies similarly, while this was a minority opinion in the other two states. Convenience stores were regarded as a natural place to sell tobacco. In each state, it was regarded as normal and commendable for some stores to want to stop selling tobacco, although few participants could imagine convenience stores doing so. Views on government's role in setting limits on tobacco sales varied, with California and New York participants generally expressing support for restrictions, and Ohio participants expressing opposition. However, even those who expressed opposition did not approve of tobacco sales in all possible venues. Banning tobacco sales entirely was not yet normative.

**Conclusion:**

Limiting the ubiquitous availability of tobacco sales is key to ending the tobacco epidemic. Some limits on tobacco sales appear to be normative from the perspective of community members; it may be possible to shift norms further by problematizing the ubiquitous presence of cigarettes and drawing connections to other products already subject to restrictions.

## Introduction

Denormalizing tobacco use through social norm change that “pushes tobacco use out of the charmed circle of normal, desirable practice” [1], p. 225 has been a key component of tobacco control, motivating smokers to quit [2] and protecting nonsmokers from second-hand smoke [3]. Yet, in the U.S., tobacco products remain ubiquitous, available 24 hours a day, 7 days a week from numerous retail outlets, helping to sustain the ongoing tobacco epidemic [4], p. 307. Some localities have limited the number and type of retailers that sell tobacco [5]–[6], and some retailers have voluntarily stopped selling tobacco altogether [7]–[9], including most recently CVS, the national pharmacy chain [10]. These developments may foreshadow the beginning of the denormalization of retail tobacco sales. Recently, the U.S. Surgeon General's Report on The Health Consequences of Smoking identified restrictions on sales as one of two promising “endgame” strategies for the tobacco epidemic [11].

Understanding community norms surrounding tobacco sales can help accelerate this process, by identifying aspects of tobacco sales on which denormalization efforts might most productively focus. A handful of studies have examined public opinion regarding tobacco sales in various venues, particularly pharmacies and grocery stores [12]–[15]. These showed that slightly more than half of San Franciscans (56%) and New Yorkers (57%) sampled favored banning tobacco sales in pharmacies [12], [14], and half of New Yorkers favored banning sales in grocery stores [12]. Support for both policies is lower nationally [15].

This study draws on focus group discussions among customers of California, New York, and Ohio retailers that had voluntarily ended tobacco sales to examine normative assumptions concerning where cigarettes should or should not be sold, voluntary decisions to discontinue tobacco sales, and government limits on such sales.

## Methods

From 2009–2013, we conducted 15 focus groups (moderated group interviews useful in exploring variability in poorly understood phenomena) [16]–[17] with 84 patrons of California, New York, and Ohio grocery stores and pharmacies that had voluntarily discontinued tobacco sales in the seven years prior to data collection (table 1). We identified these businesses through media accounts, information obtained from tobacco control organizations and departments of public health, and telephone inquiries to grocery stores and pharmacies; details have been previously published [7], [9].

**Table 1:** Focus group participant characteristics (N = 84).

	FG 1	FG 2	FG 3	FG 4	FG 5	FG 6	FG 7	FG 8	FG 9	FG 10	FG 11	FG 12	FG 13	FG 14	FG 15
Location	N. CA	N. CA	N. CA	N. CA	N. CA	N. CA	S. CA	S. CA	NY	NY	NY	NY	NY	OH	OH
Participants (n)	7	8	6	7	6	8	6	2	7	5	3	2	2	6	9
Age range	29–63	38–70	26–55	20–68	18–68	20–79	33–45	24	24–57	23–54	39–65	28–37	34–59	30–73	22–76
Women	4	6	1	6	3	4	0	2	4	4	2	0	1	3	6
Men	3	2	5	1	3	4	6	0	3	1	1	2	1	3	3
Asian	2	3	1	1	0	1	1	0	0	0	0	0	0	0	0
African American	0	0	3	0	0	5	0	0	1	0	1	0	0	1	0
Hispanic	0	0	1	1	0	0	0	0	0	1	0	0	0	0	0
Other*	1	1	0	0	1	1	0	0	0	0	1	0	0	0	0
White**	4	4	1	5	5	1	5	2	6	4	1	2	2	5	9
Current tobacco users	3	1	2	0	0	2	1	2	3	2	1	2	0	3	4
Former tobacco users	2	4	1	6	4	2	1***	0	2	0	1	0	1	2	2
Never tobacco users	2	3	3	1	2	4	3	0	2	3	1	0	1	1	3

* Other = American Indian or Alaskan Native, multiracial, Native Hawaiian or Pacific Islander, or other.

**White =  Non-Hispanic white.

*** 1 member of this group had missing data for former tobacco use.

The study was approved by UCSF's Committee on Human Research, IRB #10-00850. We recruited participants by posting ads on Craigslist, a classified ads website; we also sent flyers to community centers and libraries for posting, and to members of tobacco control organizations, who agreed to post flyers in or near tobacco-free retailers on our behalf. (For businesses in the San Francisco Bay Area, we posted flyers ourselves in or near tobacco-free retailers.) Eligibility requirements were age 18 and above, ability to speak and read English, and patronage of a particular tobacco-free retailer. Participants called a toll-free number and were screened to ensure that they met these requirements. See table 1 for information on the demographics of group participants.

Focus group sessions typically took place in meeting rooms at public libraries located near the particular tobacco-free retailer under study. They lasted 1.5 hours. After a brief introduction, participants read and signed a consent form and agreed to audiotaping. The discussion was moderated by one of three experienced researchers, including the first author, using a low moderator involvement approach (i.e., open-ended questions and minimal moderator-initiated direction) [17]. One other researcher was also typically present, sitting with participants, taking notes, and occasionally asking clarifying questions. Questions focused on participants' opinions about why a retailer would voluntarily end tobacco sales, characteristics of retailers that might choose to do so, and potential impacts of the decision, as well as participants' support for voluntary versus mandatory policies governing tobacco sales. Participants also completed a short survey that included demographic and tobacco use questions. Participants were compensated $40.

Audiotapes were transcribed by a professional transcribing service. The first author checked the transcripts for accuracy, then coded them through a collaborative, inductive process involving data review and discussion of key points with two other researchers. We created an initial set of codes collectively; as data review progressed, we refined and added codes, re-coding earlier transcripts to reflect changes. We used the software package NVivo9 for data management [18]. Both authors analyzed and interpreted the data using qualitative content analysis, which involves identifying themes or patterns in systematically coded text [19]. We chose quotes that were illustrative of the themes we identified. We identify speakers as male (M) or female (F), a current tobacco user (CTU), former tobacco user (FTU), or never tobacco user (NTU), and identify their particular focus group by number (e.g., FG1).

## Results

### Norms regarding where cigarettes are/should be sold

Participants considered cigarettes to be more or less integral to the identity of certain types of retailers, with California residents making the most distinctions. Participants in California focus groups generally agreed that it was inappropriate for pharmacies to sell tobacco products “because they're supposed to promote health” (F-CTU, FG1). Several noted the contradictions between a pharmacy, which they saw as akin to a “doctor” or a “hospital” selling medicines to “cure people” while also “selling poison” that “ruin[s] people's lives” or “kills you” (M-NTU, FG7; M-CTU, FG3; F-NTU, FG5). One participant explained that “if I was a pharmacist, I wouldn't want to practice … in the same business that sold junk food and tobacco and alcohol … because it seems contradictory” (F- NTU, FG2). It seemed “logical” then for pharmacies to choose not to sell tobacco products (F-NTU, FG5). Some New York and Ohio focus group participants shared this view, but it was typically the minority opinion in these groups. Instead, these groups saw no particular difference between pharmacy and grocery store tobacco sales. As one focus group participant explained, “I think the issue is still just the cigarettes. I don't think what else [is] in the store is really affecting [it]” (M-CTU, FG12). A voluntary end to tobacco sales in a pharmacy – particularly, a large, chain pharmacy – was significant, if at all, for its impact on tobacco product accessibility, because “Rite Aid and CVS are almost on every single corner” (F-NTU, FG15). If they were to end sales, smokers would have fewer places to buy cigarettes, and, according to some participants, “might be upset” (F-NTU, FG15].

Grocery stores had a more complex relationship to tobacco sales. Although some focus group participants in all three states characterized grocery stores as “one stop shops” for convenient purchase of both food and tobacco (M-NTU, FG7), smokers (or nonsmokers with friends who smoked) pointed out that few people actually purchased tobacco at grocery stores because it was “really expensive” (F-NTU, FG9; F-FTU, FG14; F-CTU, FG14; F-CTU, FG1; M-CTU, FG3). Smokers also pointed out that it was actually inconvenient to purchase both tobacco and food at grocery stores, because it typically involved “two steps”: shoppers had to stand in one line to purchase food and another (at the customer service counter) to purchase tobacco (F-FTU, FG13).

Regardless of their perspective on the convenience of purchasing tobacco in grocery store, focus group participants in all three states typically did not associate tobacco sales with grocery stores that sold organic, “fresh”, or other “healthy” products because, much like pharmacies and tobacco, those foods and tobacco didn't “go together” (F-CTU, FG1; F-NTU, FG15; F-NTU, FG10; F-NTU, FG4; M-FTU, FG2). Many participants (including smokers) were surprised to learn that the “healthy” grocery store they patronized had, until relatively recently, sold tobacco products, because, they said, doing so was inconsistent with the stores' apparent focus on healthy living. They also explained that they had never noticed tobacco products for sale at these stores in the past, due, in part, to a lack of in-store advertising; as one focus group participant pointed out, he had never seen “a big Marlboro sign” at his grocery store when it still sold tobacco (M-NTU, FG10).

There was near universal agreement, however, that cigarettes were integral to the identity and perhaps the continued existence of convenience stores. Most participants could not imagine a convenience store NOT selling tobacco products, given how many shoppers bought cigarettes at such stores: “It's almost unthinkable. I just think that convenience stores wouldn't be around if they weren't selling cigarettes” (F-NTU, FG2). If a convenience store were to end tobacco sales, it would “lose a lot of money” (F-FTU, FG 11) or “have a going out of business sale” (F-CTU, FG1). Typically, at least one person in each group stated that smokers would be “upset”, (M-FTU, FG14; M-CTU, FG12; F-CTU, FG10; F-CTU, FG8; M-NTU, FG7; F-CTU, FG6) “indignant” (M-NTU, FG13) or possibly even “riot”(F-NTU, FG15) if a convenience store stopped selling tobacco products. Several went further, arguing that convenience stores “should” sell cigarettes:

Convenience stores should sell cigarettes because that's your quick stop, and you go in there and get what you need and you go out.

Facilitator: But may I ask why you used the word “should”— they “should” sell cigarettes?

Because that's where you go for such things, and you just know you go in there and you get your coffee and your newspaper—if anyone still reads newspapers—and your cigarettes. It's the American way. (F-NTU FG4)

In contrast to other focus group members who thought that convenience stores ought to sell tobacco products if they wanted to stay in business, this participant explicitly suggested that they should sell tobacco because people expected it.

In discussing where cigarettes might or might not be sold, focus group participants also drew connections between neighborhood types and the likelihood of cigarette sales. Residents of predominantly poor or minority neighborhoods in California pointed out that nearly every corner featured a convenience store selling tobacco:

We can literally walk from one store . . . One closes at 4:30. … We have the schedules down pat. And there's another store: If you want your cigarettes before 11:00 then you have to go there. And if not, if you miss the 11:00, then there's another one up the street. (F-CTU, FG6)

To “walk to [a] corner store and [find] they didn't sell tobacco” was simply a “dream” for some members of these communities (M-NTU FG3). In addition, all but one focus group agreed that retailers who chose to no longer sell tobacco would likely be located in wealthier communities, as highlighted in this exchange among FG7 participants:

Speaker #1: I think [a retailer who ends tobacco sales] is not going to be [in] a real low-end area. … (M-NTU)

Speaker #2: There's probably a lot more [tobacco] sales in the lower end areas. … So they'd be hurt more by not selling [tobacco] (M-NTU).

Speaker #3: Yeah, … it would not be in the inner cities. It'd be [affluent places] like, … Woodland Hills or Brentwood or Beverly Hills, … Pacific Palisades, people more upper class (M-NTU).

Indeed, studies have shown that tobacco retailers are concentrated in economically and socially deprived communities, and in neighborhoods with high proportions of African Americans and Hispanics [20]–[25].

### Ending tobacco sales voluntarily is a normal and commendable practice

California, New York and Ohio focus group participants could easily imagine that retailers might choose to discontinue tobacco sales, with such a decision seemingly in the range of accepted practice. They offered a variety of reasons why a retailer might do so, ranging from the pragmatic to the idealistic; most were consistent with reasons offered by retailers themselves [7]–[9]. Pragmatic reasons for voluntarily ending tobacco sales included declining tobacco sales; avoiding the regulations or other “hassles” associated with tobacco sales (i.e., checking id, controlling access, facing censure for getting caught selling tobacco to minors, or being drawn into future lawsuits against the tobacco industry); responding to community pressure or local government incentive; or trying to enhance the store's image (e.g., by appealing to parents or health-conscious customers, or differentiating the store from larger corporate-owned chains). Idealistic reasons included retailers' concern for the health of the community; personal experience with a tobacco-caused disease; religious beliefs; a desire to prevent children's exposure to tobacco advertising; or “guilt” over their association with the tobacco industry. Despite California participants' view of pharmacies and tobacco as incompatible for health reasons, like their counterparts in other states, they did not consider pharmacies more likely than grocery stores to end tobacco sales for idealistic reasons.

Retailers' decision was also “normal” in the sense that it would most likely maintain the status quo for the business itself. Most focus group participants believed that customers who were smokers would continue to shop at tobacco-free retailers because tobacco products were unlikely to be the primary draw. Focus group members also argued that the new policy was unlikely to attract many new customers; they assumed that most people were unaware of the policy (just as many focus group members were), or, if people were aware, would not base decisions about where to shop on a store's tobacco-free status. As one California focus group participant explained, “I think most people are practical. They go to the store they feel comfortable with, that's close to them. All these things that are pluses … like if cigarettes are gone, they either don't know about it or they don't really care” (F-FTU, FG2).

Asked about the possible impact on smokers of a retailer voluntarily ending tobacco sales, participants typically focused on the inconvenience of having to shop elsewhere for cigarettes. However, most groups did not consider this to be especially burdensome, as smokers were “used to being inconvenienced” (M-NTU FG2). “What are you going to do? You're going to go somewhere else, to another store that does have [cigarettes]. So I don't think it's that big of a deal” (F-CTU, FG6).

Nearly everyone regarded retailers' decision to discontinue tobacco sales as commendable. Many focus group participants saw the decision as good for public health in general, noting, for example, that “any institution or place that sells less poison is … good for the overall public” (F-FTU, FG2). Another participant saw the decision as a choice for the “greater good”, because “all of society pays for the burdens of people who are sick or afflicted” by tobacco-caused disease (F-NTU FG10). Deciding to end tobacco sales was seen as reflecting business owners' concern for customers' health. These businesses were “not taking advantage of smokers who are addicted” (F-NTU, FG9); they cared “more about health than making money” (F-NTU, FG4). Among the handful of people who did not voice support, most had no opinion, or expressed indifference, viewing it as a “nice gesture” that was not “particularly meaningful” (M-NTU, FG7). Only a small number were opposed, believing that it would spark a trend that would make cigarettes harder to find and more expensive, or rejecting the policy as overly paternalistic.

### Government limits on tobacco sales are (mostly) a normal and commendable practice

Most (11 of 15) focus groups supported laws like those passed in San Francisco and several Massachusetts localities prohibiting pharmacy tobacco sales. Some participants saw such laws as leveling the playing field for all pharmacies, preventing pharmacies that voluntarily chose to stop selling tobacco from losing customers to pharmacies that continued to sell. Others thought these laws would make it harder for kids and adults to obtain tobacco, “kind of like raising the taxes on [cigarettes]” (F-CTU, FG8), and thereby have a broader impact on smoking prevalence than strictly voluntary policies. Smokers in these groups also supported mandatory policies partly because they knew they would not impede their ability to buy tobacco; one explained that “if there's prohibition lurking, then I'll have a different tune to sing to you. But I like it because it protects the youth” (M-CTU, FG7). These groups also agreed with the broader proposition that it made sense for governments to determine where cigarettes were sold, making comparisons to existing laws governing alcohol sales or public place smoking with which they were familiar and comfortable.

In four focus groups, two in California and two in Ohio, opposition to laws prohibiting pharmacy tobacco sales dominated discussions, primarily based on “slippery slope” fears. For example, one California smoker stated that

I don't like it. I think it's infringing on my personal choice, and my freedom, and … pursuit of happiness….First they're starting with pharmacies, then after that's rolled in and everyone's accepting that, they're going to start doing more, grocery stores and convenience stores and everything. San Francisco is a trend-maker, and … I believe that's what they're going to … do. (F-CTU, FG1)

Ultimately, she feared, smoking itself would be outlawed. An Ohio participant asked “what's the next ban going to be?” (F-NTU, FG 15), and another suggested it would be a ban on “16 ounce or whatever it is” sodas, as proposed in New York (F-CTU, FG15). Another commonly voiced objection was the belief that such a law “infring[ed] on … public rights” (M-FTU, FG1) or was an example of “the government sticking their nose in and taking more of our rights away” (F-NTU, FG15). These groups thought that the decision to sell or not to sell tobacco should rest entirely with the retailer, as illustrated in the following discussion among participants of FG15:

Moderator: Does it make sense to have some sort of law or regulation that says you can sell tobacco here, but not here? Or should it just be completely up to the retailer?

Speaker #1: Retailer (F-NTU).

Speaker #2: Retailer (M-CTU).

Speaker #3: I'm going with the retailer, yeah (F-CTU).

Speaker #4: Retailer (F-NTU).

Moderator: Okay. So, if you want to sell it in a furniture store, you should —

Speaker #4: That's up to the business.

Speaker #2: I believe it should be up to the business.

Speaker #4: You have a right to sell whatever is legal in a business.

Moderator: All right. The baby clothes store, they could sell?

Speaker #4: If they wanted to sell cigarettes, it doesn't mean I'll shop there, but that's their right to sell them.

Speaker #2: That's a bit of a stretch! I don't think they would have many customers.

Speaker #4: But it's their right to decide as a business if they want to do it or not.

Even within this discussion in which participants supported the idea that there should be no government limits on tobacco outlets, community norms regarding what type of product cigarettes were and where they should and should not be sold were implied. The speakers' responses suggested that cigarettes and baby things were, in fact, incompatible products to sell together, and that a baby clothes shop breaching this norm might risk failure.

### Banning tobacco sales is not a community norm

The idea of a community banning tobacco sales entirely typically brought much more mixed responses within focus groups (although 3 groups – 2 in California and 1 in New York — were uniformly enthusiastic about the idea, and 2 – both in Ohio — were uniformly opposed). For many, whether they supported the idea or not, it was impossible to imagine a community banning tobacco sales. Some simply asserted that it “sounds like a fairytale” (F-FTU, FG2) or was “not possible” (F-NTU, FG2). Others offered reasons why a tobacco sales ban was unimaginable, including a sense that a ban was an extreme step that had the potential to create “chaos” or “anarchy” (F-FTU, FG4). For example, a smoker stated that “because … nicotine is so addictive, it just doesn't seem realistic that they could just completely ban the selling of tobacco products. … I think a lot of people would be running around, you know, complete chaos. And it just wouldn't be a pretty sight” (M-CTU, FG9). Others thought that a ban would “never happen” because governments made too much money from tobacco taxes or because politicians relied on tobacco industry campaign contributions (F-FTU, FG4; F-FTU, FG9).

Some focus group participants were willing to accept that a community *might* ban tobacco sales, but expressed reservations about such a step, believing that a ban would not work as intended, or that it should be introduced gradually, after a “national dialogue” (F-NTU, FG3). Among those who saw a ban as ineffective, several drew parallels with the failure of alcohol prohibition in the 1920s to curb alcohol consumption, or simply asserted that people would “still smoke” (M-NTU, FG3) finding “a way to get around [the law] in one way, shape, or form” (M-NTU, FG8).

Those who expressed unmitigated support for the idea of a community banning tobacco sales tended to be nonsmokers; several justified their support by drawing parallels to the acceptability of banning illicit drugs. For example, one participant noted that “we're not allowing heroin to be sold” (F-NTU, FG9), while another argued that “the little disclaimer on the [cigarette] packs about ‘[smoking] causes this and this and this,’ that doesn't seem to sway anybody. … You have to force them for the greater good just like drugs” (M-NTU, FG13). Those who rejected the idea of a tobacco sales ban were a mix of smokers and nonsmokers; the reasons for their opposition centered on a dislike of “big government”, a denial of “choice”, or a conviction that a ban would lead to black markets and possibly violence.

### Limitations

This study has several limitations. Focus group members were a self-selected, non-representative sample; thus, our findings cannot be generalized to all customers of retailers who voluntarily ended tobacco sales. Focus groups were also conducted in only three states, with approximately half of the groups based in California. Our choice of states was influenced both by our awareness of particular retailers who had ended tobacco sales voluntarily, and the willingness of those retailers to be interviewed. Thus, customers of tobacco-free retailers in other states may have different perspectives.

## Discussion

This study suggests that there are existing community norms regarding where cigarettes should or should not be sold, even in states with very different political climates regarding government regulation. The notion that cigarettes should not be sold in stores focused on healthy products was strongest in California, where focus group participants considered both pharmacies and grocery stores selling organic foods to be incompatible with selling cigarettes. However, in New York and Ohio, pharmacy sales were not consistently regarded as violating community norms. Since a recent, hard-fought battle to ban cigarette sales in all San Francisco pharmacies garnered considerable media coverage, it may be that the vigorous debates around this policy initiative, including coverage of the tobacco industry lawsuit challenging it, shifted opinions in California. California residents already have been exposed to two decades of mass media campaigns aimed at changing views of both smoking and the tobacco industry [26], and generally have negative perceptions of tobacco companies [27]. In addition, California has the second lowest adult smoking prevalence in the United States [28], which itself influences broader community norms about tobacco use.

Even when participants argued against any government limits on tobacco sales, they made distinctions indicating that cigarettes were not appropriate products to sell everywhere. This suggests that targeted media campaigns problematizing the ubiquitous presence of cigarettes, especially in stores frequented by children or selling products for children, may be helpful in shifting norms and consistent with community expectations.

In each state, it was regarded as “normal” and acceptable for some stores (particularly pharmacies and grocery stores) to want to stop selling tobacco. Participants were able to readily identify and support possible rationales for doing so, including contributing to public health and to the image of the stores as retailers who cared about their customers. Even smokers indicated acceptance of such voluntary decisions. However, for convenience stores to stop selling tobacco was widely regarded as improbable, even unimaginable at this point. If jurisdictions wish to work to reduce the number and density of tobacco outlets, starting with pharmacies and grocery stores seems the most expedient route. In addition to likely community support, these retailers may be the most receptive to removing tobacco from their shelves. Pharmacists have long favored ending tobacco sales [13], [29]–[34], and CVS' decision to do so (and the positive press it has generated for the company) [35] can be held up as a model. For grocery stores, tobacco may no longer be a major profit item [7], [9]; owners may be readily persuaded to replace it with a potentially more lucrative product. By contrast, community acceptance of tobacco sales in convenience stores, coupled with such stores' reliance on tobacco sales to generate profits [36] and strong relationship with the tobacco industry [37], [38], may engender more opposition to efforts to limit their sales.

Views on government's role in setting limits on where cigarettes could be sold varied by state, with the more pro-regulatory politics of California and New York and the more anti-regulatory politics of Ohio reflected in group discussions. As with other tobacco control policies [39], states with pro-regulatory climates are likely to take the lead on addressing the retail sales of cigarettes, with others following (if at all) after the policies have been tested in several states and survived any legal challenges from tobacco companies. For example, limiting tobacco sales to state-run outlets, as has been done with alcohol in some states [40] may be an acceptable extension in such states.

Banning tobacco sales entirely is not yet normative. This finding is not surprising, as it is very rare that any public health or government official has suggested such a thing, and to do so has in the past been to court censure [41]. Focus group participants for the most part could not even imagine it. However, some did suggest that after more public “dialogue”, such a step might be introduced in a phased way. As the U.S. moves toward planning for a tobacco endgame, the idea of “phasing out cigarettes” may be a way to shift social norms by comparing cigarettes with other deadly products that have been phased out of use. Fear of extreme reactions (“riots”) has been raised in conjunction with the introduction of almost every important tobacco control policy to date, and has never materialized, partly because most smokers want to quit. There is emerging evidence that smokers support such policies because they help them stop smoking [42].

## Conclusion

Excluding tobacco sales from the “charmed circle” of desirable, socially acceptable retail practice and thereby limiting the ubiquitous availability of tobacco products is key to ending the tobacco epidemic [4]. Some limits on tobacco sales appear to be normative from the perspective of community members; others may become so by drawing connections to products that are already subject to restrictions or that have been eliminated entirely. The “national dialogue” about how to bring the tobacco epidemic to an end should include discussion of how to further reduce the number of tobacco retail outlets.

## Supporting Information

Text S1 **California retailers study.**
(PDF)

Text S2 **New York and Ohio retailers study.**
(PDF)
