# Supplementary material for: Understanding Community Norms Surrounding Tobacco Sales
Source: PLoS One. 2014 Sep 2;9(9):e106461. doi: 10.1371/journal.pone.0106461 (PMC4152285; doi:10.1371/journal.pone.0106461)
Supplement: Text S1 — California retailers study. (PDF) [file pone.0106461.s001.pdf]

McDaniel and Malone *BMC Public Health* 2011, **11**:848
http://www.biomedcentral.com/1471-2458/11/848

BMC Public Health

RESEARCH ARTICLE Open Access

Why California retailers stop selling tobacco products, and what their customers and employees think about it when they do: case studies

Patricia A McDaniel* and Ruth E Malone

Abstract

Background: In California, some 40, 000 retailers sell tobacco products. Tobacco's ubiquitousness in retail settings normalizes use and cues smoking urges among former smokers and those attempting cessation. Thus, limiting the number of retailers is regarded as key to ending the tobacco epidemic. In the past decade, independent pharmacies and local grocery chains in California and elsewhere have voluntarily abandoned tobacco sales. No previous studies have examined the reasons for this emerging phenomenon. We sought to learn what motivated retailers to discontinue tobacco sales and what employees and customers thought about their decision.

Methods: We conducted case studies of seven California retailers (three grocery stores, four pharmacies) that had voluntarily ceased tobacco sales within the past 7 years. We interviewed owners, managers, and employees, conducted consumer focus groups, unobtrusively observed businesses and the surrounding environment, and examined any media coverage of each retailer's decision. We analyzed data using qualitative content analysis.

Results: For independent pharmacies, the only reason given for the decision to end tobacco sales was that tobacco caused disease and death. Grocers listed health among several factors, including regulatory pressures and wanting to be seen as "making a difference." Media coverage of stores' new policies was limited, and only three retailers alerted customers. Management reported few or no customer complaints and supportive or indifferent employees. Pharmacy employees were pleased to no longer be selling a deadly product. Grocery store management saw the decision to end tobacco sales as enhancing the stores' image and consistent with their inventory of healthy foods. Focus group participants (smokers and nonsmokers) were largely unaware that retailers had stopped selling tobacco; however, almost all supported the decision, viewing it as promoting public health. Many said knowing this made them more likely to shop at the store. Most thought that advertising the store's policy was essential to generate good public relations and tobacco norm changes.

Conclusions: Voluntary retailer abandonment of tobacco sales both reflects and extends social norm changes that have problematized tobacco in California. Our findings suggest that such voluntary initiatives by retailers are welcomed by consumers and should be publicized, enhancing public health efforts.

Background

Approximately 40, 000 California retailers sell tobacco products [1]. Outlet density increases the likelihood of smoking among minors and adults [2,3], due partly to tobacco advertising in tobacco outlets, which normalizes and promotes tobacco use [4-10] and, among smokers and former smokers, triggers smoking [9,11-13]. The ubiquity of tobacco outlets also normalizes purchase and sale of addictive and deadly products [14]. The Institute of Medicine regards limiting the number of tobacco outlets as key to ending the tobacco problem [[15], p. 307].

Recently, however, some retailers have begun voluntarily abandoning tobacco sales, despite the absence of any external policy mandate. Independent pharmacies have led these initiatives, followed by local grocery chains. But why they have done so has never been explored. Previous research on ending tobacco sales has

* Correspondence: patricia.mcdaniel@ucsf.edu
Department of Social and Behavioral Sciences, School of Nursing, University of California, San Francisco, 3333 California Street, Suite 455, San Francisco, CA 94118, USA

BioMed Central

© 2011 Patricia A McDaniel; licensee BioMed Central Ltd. This is an Open Access article distributed under the terms of the Creative Commons Attribution License (http://creativecommons.org/licenses/by/2.0), which permits unrestricted use, distribution, and reproduction in any medium, provided the original work is properly cited

focused exclusively on pharmacies, and has only examined the policy climate and characteristics that distinguish tobacco-free pharmacies from those that sell tobacco [16-26]. Using a case study approach that included interviews with business owners and employees, customer focus groups, and in-person observations, we explored why California retailers are abandoning tobacco sales and what consumers and employees think about the decision to do so.

Methods

We used several methods to identify a set of 11 California grocery stores and pharmacies that had stopped selling tobacco products within the past 7 years. A search of the Lexis Nexis and Newsbank databases for newspaper articles referencing California retailers that had voluntarily discontinued tobacco sales yielded 3 eligible grocery stores and 1 pharmacy. A list provided by the Los Angeles County Department of Public Health of county pharmacies that had stopped selling tobacco in 2008 or earlier yielded 4 eligible pharmacies.

To identify additional retailers, we telephoned pharmacies and grocery stores to ask whether they sold tobacco products and, if not, when they had stopped. We gathered pharmacy contacts from the State of California's Department of Consumer Affairs website [27]. We called all currently licensed pharmacies in 27 counties (excluding counties in the far east and far north of California and pharmacies in the cities of San Francisco and Richmond, which were already legally prohibited from selling tobacco) (N = 1, 447); because most independent pharmacies in California ended tobacco sales more than 7 years ago while most chain pharmacies continue to sell, only one was eligible. According to an online business directory, there are over 20, 000 California grocery stores [28]; given limited resources and our study's exploratory nature, we gathered 25 grocery store names based on personal knowledge and obtained locations and phone numbers from the internet. We found 2 that were eligible.

The study was approved by UCSF's Committee on Human Research. Of 11 eligible retailers, 7 (64%) agreed to participate (4 pharmacies, 3 groceries) after being contacted by phone by the first author. We agreed not to reveal in publications the names of the business or anyone we interviewed. Three of the four non-participating retailers were similar to participating retailers in terms of location, size, and product types. One non-participating retailer was unique in that it was a "big box" chain store.

For each retailer, we obtained informed consent and conducted an in-person or telephone interview with an owner or store manager; these individuals were typically most involved in creating and/or implementing the voluntary tobacco-related policy (Table 1). In three cases, we also interviewed at least one employee in order to obtain the perspective of staff who were more likely to have direct experience with customers and with selling vs. not selling tobacco (Table 1). Given that so little is known about retailers' voluntary decision to end tobacco sales, interviews were ideally suited to gather detailed information about this emerging phenomenon [[29], p. 4]. Questions explored why and how the tobacco-free policy was implemented, its perceived impact on business, customer reaction, and the interviewee's satisfaction with the policy. All but two of the interviews were audiotaped (one respondent declined and a second granted an interview before the audiotape equipment was accessible; the interviewer documented these using written notes). There were no discernible differences in quality between face-to-face and telephone interviews. To protect businesses' identities, we numbered the businesses (e.g., "grocery 1"). We refer to interviewees by job title and, when we interviewed more than one person in the same job title, by number (e.g., "employee 1").

For each participating business, we also conducted a customer focus group (in one case, due to over-recruitment, we conducted 2). We recruited by posting on Craigslist, a classified ads website, and, for Northern California businesses, by posting flyers at community centers, libraries, and near the business. Eligibility requirements were age 18 and above, ability to speak English, and patronage of the particular business in our study. Focus groups were moderated by an experienced researcher using a low moderator-direction approach [30]. We obtained signed consent; participants were told that their names would not be used in publications. Open-ended discussion questions explored customers' knowledge of the store's tobacco-free policy and attitudes towards it. Participants also completed a short sociodemographic questionnaire that included measures of current and former tobacco use (yes/no). Participants were compensated $40. See Table 2 for focus group details.

We conducted unobtrusive observations at each store (for groceries, we randomly selected one store in the chain), noting product types, signs advertising the tobacco-free policy, and the number of tobacco retailers within a three-block radius. Finally, we searched media databases (Access Newsbank, Lexis Nexis, and Google news archive) to determine the extent of media coverage. Triangulation of data (interviews, focus groups, observations, and media analysis) provided cross-data validity checks [[31], p. 248].

Interview and focus group transcripts were transcribed and checked for accuracy, then coded through a collaborative, inductive process involving data review and

Table 1 Participating grocery stores and pharmacies

Name	Number of stores	Location	Median household income rank of neighboring community (0-99)*	Interviewees (*N* = 17)
Grocery 1	4	Northern California	82-99	Owner; 4 store managers
Grocery 2	9	Northern California	82-96	Owner; 1 store manager
Grocery 3	2	Northern California	96-98	Owner; 1 employee
Pharmacy 1	1	Northern California	76	Owner; 3 employees
Pharmacy 2	Part of national chain	Northern California	94	Store manager
Pharmacy 3	1	Southern California	99	Owner
Pharmacy 4	1	Southern California	73	Owner; 1 employee

*From http://zipwho.com

discussion of key points. We created an initial set of codes collectively; as data review progressed, we refined and added codes, re-coding earlier transcripts to reflect changes. We used the software package NVivo8 for data management [32]. Given our interest in providing in-depth knowledge of retailers' decision to end tobacco sales, we analyzed data using qualitative content analysis, which involves identifying themes or patterns in systematically coded text [33]. We chose quotes that were representative of the themes we identified.

Results

Description of stores

All three grocery stores in our sample were Northern California chains, with 2-9 individual stores (Table 1). All sold specialty "healthy" products (e.g., organic produce and gluten-free foods) and were located in relatively affluent communities (Table 1). They also sold wine and spirits, and a wide range of high fat, salt, and sugar items. When still selling tobacco products, none of these stores reportedly offered discounts or displayed tobacco advertising. There were 3-5 tobacco retailers within a three-block radius of each store observed.

Three of the four pharmacies in our sample were independent; one belonged to a national chain whose other stores continued to sell tobacco products (Table 1). Two independents were located in isolated areas, and were the only available local pharmacies. Two (one independent, one chain) were located in affluent communities (Table 1). Only the chain pharmacy sold alcohol. There were 2-5 tobacco retailers within a three-block radius of each pharmacy.

Why retailers discontinued tobacco sales

Among the independent pharmacies, the link between tobacco and disease and death was the only reason given for abandoning tobacco sales. In one case, this link was personal, with the owner citing the loss of several family members to tobacco-related disease:

> I've always had that idea [to stop selling cigarettes]. ... My grandmother smoked. She died of ... pulmonary embolism that I think probably had a lot to do with smoking. And my mom [a smoker] died of cancer. (Owner, pharmacy 1)

In two cases, the link between tobacco and disease was more abstract, although still a compelling reason to end tobacco sales:

> We're trying to be so healthy, that's what a pharmacy is all about. ... When I started selling cigarettes as the clerk in 1960 ... we didn't know. There wasn't even the warning on the package from the Surgeon General, and so we didn't know they were bad. ... So

Table 2 Customer focus group participants

Retailer	Number of participants	Number of current smokers	Number of women	Age range	Ethnicity*
Grocery 1	7	3	4	29-63	2 A; 1 NH/PI; 4 NHW
Grocery 2	8	1	6	38-70	3 A; 1 M; 4 NHW
Grocery 2	6	2	1	26-55	1 A; 3 AA; 1 HW; 1 NHW
Grocery 3	7	0	6	20-68	1 A; 1 HW; 5 NHW
Pharmacy 1	6	0	3	18-68	1 M; 5 NHW
Pharmacy 2	8	2	4	20-79	1 A; 5 AA; 1 NHW; 1 O
Pharmacy 3	6	1	0	33-45	1 A; 5 NHW
Pharmacy 4	2	2	2	24-24	2 NHW

**A* Asian, *AA* African American, *HW* Hispanic white, *M* multi racial, *NH/PI* Native Hawaiian or Pacific Islander, *NHW* non Hispanic white, *O* other

> this is how I explained it to my staff here, was that at one time, we didn't realize that we shouldn't be selling cigarettes, but now it's more than obvious. (Owner, pharmacy 4)
>
> I was always against [selling tobacco products]. And ...with the reason ... that it causes cancer, I decided I just don't want to be part of it. (Owner, pharmacy 3)

By contrast, the chain pharmacy had discontinued tobacco sales in one particular store as a test to "see how the market fared with not having cigarettes" (Manager, pharmacy 2).

Grocery owners and managers also offered health-related reasons (both abstract and personal) for ending tobacco sales; however, all but one also mentioned at least one additional reason, such as declining tobacco sales or difficulties associated with tobacco sales, including attempts by minors to purchase tobacco and the licensure requirements:

> Normally with cigarettes, you're going to have a younger ...crowd try to buy those. ... It's one less headache. .... It was that consideration and the fact that... it's not a good healthy habit for people to have. So ..., I figure, "Okay, I'm not promoting it if I don't sell a product." (Manager 1, grocery 1)
>
> It kind of got on our radar that, uh, cigarettes were not a [briskly selling] ... product. ... We started out in the health food business. ... And then when we started acquiring grocery stores, cigarettes came, just the whole culture of the grocery store. It was embedded that you would sell cigarettes. And it was funny, because over all the years when people complain from special interest groups to me, "Why do you sell this kind of meat?" and, "Why do you sell this kind of fish?" and, "Why do you sell this?" and, "Why do you sell that?" My stock answer used to be, "If I'm going to decide what consumers should buy and they shouldn't buy at our store, and I made the call exclusively, I would start with cigarettes." I couldn't understand why someone would approach me and ask me not to sell a certain kind of veal ... but they didn't care if we sold cigarettes or not.... My father died from emphysema. ... I just got to thinking that, boy, I sure said this a lot of times over the years, and in talking with my partner, we said, "Yeah, you know what? It's our store and it's our business. If we don't want to sell them, you know, we don't have to sell them." (Owner, grocery 2)

Publicizing the new policy

Two grocery retailers alerted local media to their decision to end tobacco sales. One local newspaper wrote a front page story; the grocer also took out a full-page ad explaining the new policy as a way to "make a slight difference" in fighting cancer. In the second case, the grocery owners held a media event at one of their stores announcing the new policy; two local newspapers wrote brief articles. Among remaining retailers, only one received unsolicited local media coverage: brief mentions (1 year later) in two newspaper articles discussing a possible county ban on pharmacy cigarette sales.

Most stores (5) had phased in the new tobacco-free policy gradually, discontinuing tobacco orders and selling existing stock. During this phase-in, three retailers proactively alerted customers that tobacco sales would end. An independent pharmacy owner personally notified the small number of regular cigarette purchasers. Two chain groceries posted in-store signs, which were later removed. One owner, despite seeking media coverage of the new tobacco-free policy, explained why he did not continue in-store signage:

> I don't like advertising the fact that we don't have a product. ... We compete quite a bit with [a large supermarket chain]. So if I put it in people's face, like we don't sell cigarettes, and they're buying their cigarettes with their shopping at [a large supermarket chain], it gives us a disadvantage. (Owner, grocery 2)

Another owner said "I don't think it's necessary now. Most people understand that they're not going to find cigarettes in our store, period" (Owner, grocery 3).

Perceived customer reaction

Only one store owner perceived a loss of customers after discontinuing tobacco sales, but this was short-lived:

> I think I've gained customers now. Took awhile. ... Initially I think that there was a ... downturn in customers. But over time, the word got out. And I think it really was an advantage because people appreciated that I made that stand. So it was a good decision.(Owner, grocery 3)

The remaining stores experienced no loss of customers and no or few complaints:

> I don't think it affected business at all. ... It was an easy transition, you know. Nobody's gotten upset at us not carrying' em. ... Everything's just fine. (Manager 2, grocery 1)
>
> We had a few regular customers that would buy a carton every time they came in. ...[The new policy]

> upset them for a little while, and they ... got used to it. (Manager 3, grocery 1)
> Some of them [smoking customers] were pissed. But they understood. (Owner, pharmacy 3)

One grocery manager stated that several smoking customers expressed gratitude for the new policy, because it made it harder for them to buy cigarettes (implying that they were trying to quit smoking) (Manager, grocery 2). A pharmacy owner reported a similar outcome: although she had regularly encouraged her regular tobacco purchasers to quit smoking, two reported to her that they had done so only after she stopped selling cigarettes (Owner, pharmacy 3).

Retailers uniformly stated that non-smoker customers (or those who did not purchase cigarettes from them) had either no reaction to the tobacco-free policy or, occasionally, a positive reaction.

> There was a little bit of a response [from nonsmokers]. "We think it's great." "A bold move." (Manager, grocery 2)
> [Nonsmokers] didn't even notice. Not even a blip on the radar. (Manager 4, grocery 1)
> A few of them [nonsmoking customers] ... told me, "Good for you." (Owner, pharmacy 3)

Employee responses

Both management and employees reported that employees supported ending tobacco sales. Grocery managers stated that employees liked the new policy because it made their jobs easier (Manager 3, grocery 1; Manager 4, grocery 1), was good for customers' health (Manager 3, grocery 1; Manager 4, grocery 1), and was good for the store's "branding" or image (Owner, grocery 2). Employees of two independent pharmacies were happy to no longer sell cigarettes because doing so contradicted the pharmacy's focus on health. One employee explained:

> It was very, very embarrassing selling cigarettes here at the pharmacy. Because it's a pharmacy, and we're here to help people get better and feel better. And by selling cigarettes, it was ... a contradiction. ... Why am I selling you cigarettes? You're going to get sick. You have asthma. I mean, it's ridiculous. You're buying an inhaler. But then, later you come back and buy cigarettes from us. I don't want to do this. ... It was very hard especially when you've been here for a long time. And you get to know people. And you love the people that... come in. It was very difficult for me to do that, looking at them, especially when they had asthma. Looking at them coughing and not being able to breathe and taking inhalers or cough suppressants. And then, later on, looking at them outside smoking. ... "Why did I just [sell them cigarettes]? That's absolutely not right." So when that stopped, it was like a relief for me. ... And I was very happy not being able to do that to a person I knew. And so I felt very good. And it was weird because customers did ask us, "Well, why did you stop?" And it was ... very easy to answer them ... "Because we love you, and we want you to get better. And we're not going to sell you any more cigarettes. That's why we stopped." (Employee 3, pharmacy 1)

Management at two of the remaining pharmacies (one chain, one independent) claimed that employees were indifferent to the change (Manager, pharmacy 2, Owner, pharmacy 3). In only one case were employees opposed: one grocery employee claimed that most of his colleagues smoked, "so, if they run out of cigarettes then they have to leave the store and, you know, drive down several blocks to go to the next place that does sell them. So, they get irritated" (Employee, grocery 3).

Management satisfaction

Nearly all managers were happy with the owners' decisions to end tobacco sales, primarily because of tobacco's deadly effects. Two grocery managers explained:

> I'd rather not sell them just to make a dollar. ... The money is not worth somebody's health. So I feel more comfortable not selling them. (Manager 2, grocery 1)
> Personally, I thought it was great. ... I watched my grandfather die a slow death from smoking Camel straights since he was 14. ... It was painful to watch. (Manager, grocery 2)

One manager expressed indifference, stating that he would not object to selling tobacco products "if that's what the public wants" (Manager, pharmacy 2).

Similarly, store owners were nearly all satisfied with their tobacco-free policies, stating emphatically that they could not imagine any conditions that would lead them to a policy reversal. One pharmacy owner joked that she would consider selling tobacco products again if "somehow they found that tobacco smoking is good for you" (Owner, pharmacy 1). Only one owner expressed ambivalence: "I'm still uncomfortable in deciding for customers. I'm not happy with that. ... I'm not their priest. ... I'm not their teacher. ... I'm selling them groceries" (Owner, grocery 2). However, even he remained committed to the new policy, stating firmly: "we're not going back" (Owner, grocery 2).

Perceived impact on store image

Grocery store owners and managers asserted that ending tobacco sales enhanced the stores' "healthy" image and was a natural fit with the healthy products they sold. One owner explained that the policy "made a statement to our customers that [we] were very concerned about health" (Owner, grocery 3). A manager stated that the absence of tobacco products helped reinforce his store's strategy of "focus[ing] on things to help improve people's health and to make [grocery 1] synonymous with ... being a place where ... just by entering the building they're thinking that they're going to live to 100" (Manager 4, grocery 1).

Pharmacy management and employees universally agreed that cigarettes did not belong in pharmacies because, as one employee said, "pharmacy means health and cigarettes means unhealthy" (Employee, pharmacy 4). The decision to voluntarily end tobacco sales was in keeping with their understanding of pharmacies as health-promoting organizations.

Customers' policy awareness & response

In most customer focus groups (6), only 1-2 participants per group were aware that retailers they patronized had voluntarily stopped selling tobacco products, even when the business had advertised it. Asked how they felt about it, nearly all participants (including smokers) supported it because they saw it as promoting public health (see Table 3).

In each group, the majority stated that a store's tobacco free policy would have no impact on their shopping patterns. However, a small number stated that they would actually consider increasing their patronage (see Table 3).

The importance of advertising

We asked focus group participants if they thought it was important for retailers to advertise their tobacco-free policies internally, with a sign on the door or inside the store. In two groups, most participants thought it was not important to advertise since it was unlikely to alter customers' smoking habits or shopping frequency. In other groups, however, most participants thought advertising the policy was important. First, participants suggested that not advertising was a wasted public relations opportunity: "Why do it if you're not going to tell everybody? I mean, presumably, you're doing it to build your name" (Male #3, nonsmoker, pharmacy 3 focus group). Building the store's name could bring in more customers:

> ... I think why would you *not* want to [advertise], because it defeats the purpose of why you did it to begin with. .... So no, they want to promote it, I would think. And the word will hopefully spread. And people will like [it], you know–because the majority of people don't smoke to begin with. (Male #4, smoker, pharmacy 3 focus group)

Table 3 Selected consumer focus group comments

Attitudes towards retailers voluntarily ending tobacco sales	Potential impact on shopping patterns
I think it's a great idea. ... It's making a statement to the community that they're health conscious. They're looking out for the people in the community. And, maybe they'll make a precedent for other people and other stores to follow them. (Male #1, nonsmoker, pharmacy 3 focus group)	I like the idea. [It] made me wanna shop there more. ... I lost a brother to stage four lung cancer in the last 2 years because he smoked for 35 years. And so for me, it really strikes a chord. ... I really can support, and I like, their choice not to sell cigarettes. (Female #3, nonsmoker, grocery 1 focus group)
I think that it's a good thing if they volunteer, because it's really a health issue. And I know I might be contradicting myself, I am a smoker. But I also recognize that it's a healthy thing, and there's plenty of other places if people wanna get them. But teenagers and new smokers find it easily accessible. And the main issue is the kids, because they can slide into the store or the drugstore and get' em very easily. So, I know it's contradicting, but I agree with that. I think it's a very good decision. (Female #6, smoker, grocery 1 focus group)	I think it's excellent. I mean, I commend them for that, and other stores should follow. ... I wished I had known. You know, I think that's a good thing to advertise. ... Because, it makes you want to, you know, go there more. You're like, they're taking the right stand, all right, I'll support them even more, you know? (Female #2, nonsmoker, grocery 1 focus group)
They're promoting wellness, ... taking care of yourself and staying healthy and not smoking, and awareness of lung cancer, lung disease. A lot of people are dying from this disease. (Male #5, nonsmoker, pharmacy 3 focus group)	You get a positive feeling about the management. ... I would encourage them with my business to reward them for the choice they made. (Female #4, nonsmoker, grocery 3 focus group)
I like it just from the gut feeling that the fewer places where tobacco is available, the harder it is for people to access it, and the more likely some people will become discouraged and either smoke less or quit. (Female #7, former smoker, grocery 2 focus group 1)	I'd be more likely to go to [grocery 2]. ... I could go to [grocery 2] just to make a statement, a small statement that says they're not going to sell tobacco, and I support them for eliminating the sales of tobacco. (Male #6, former smoker, grocery 2 focus group 1)
I think it's a good policy to not sell tobacco in a store. ... It puts out a positive image for children. (Female #1, smoker, pharmacy 4 focus group)	I would be more inclined to shop there as well just because ... they're discouraging the smokers to smoke. (Female #2, nonsmoker, grocery 3 focus group)

Others saw advertising the policy as an important means of stimulating change, or at least promoting conversations about smoking issues and the tobacco industry:

> If you don't put the word out and educate people and advertise it, then it's not effective. People are in the dark. They can't change if they're in the dark, if they're ignorant. So I think information is major. (Female #7, former smoker, grocery 2 focus group)
>
> A parent could say, "Look. They're not selling it. You know why?" It's kind of an instrument ... to say, "Hey, you know, this is why they're not doing it." (Female #3, nonsmoker, grocery 3 focus group)
>
> It makes you have another voice saying no to the industry. But it's not individual anymore. It's a company who can make a profit, but they chose not to. So the voice is a little bit louder, in a sense. And if you have a sign, then it seems redundant, but then it also makes you question more: "Why smoking? Why they do this?" Right? So it creates more impact on people's choices. And then, "Is cigarettes really a good thing to do? ... Smoking, is it good or not?" It makes you question more, in a more subtle, but more subliminal way, too. (Female #8, former smoker, grocery 2 focus group 1)

This discussion highlights the potential value of retailers' decisions in denormalizing tobacco use–removing it from the "charmed circle of normal, desirable practice" [[34], p. 225]–and separating the tobacco industry from the community of legitimate businesses.

Limitations

Our study has several limitations. Given our sampling strategy, results are not statistically generalizable. Our study focused solely on California, a state with a strong tobacco control program that has facilitated changes in social norms around tobacco and tobacco use [35-37] and led to the nation's second-lowest smoking prevalence [38]; customers in other states might have more negative reactions to retailers ending tobacco sales. Our affiliation with a health sciences university may have resulted in a response bias among interviewees, leading them to over-emphasize the role of health in their decision to end tobacco sales. Our study offers limited insight into decision making by chain pharmacies; although our cases included a store belonging to a chain pharmacy, we were unable to interview a representative of its corporate owners. Finally, businesses in our study were not located in low income neighborhoods. Businesses with a less affluent customer base may face different economic pressures that will influence whether and how a decision to end tobacco sales is reached and how customers perceive it.

Discussion

Reducing the availability of tobacco is an important means of reducing tobacco consumption and encouraging smoking cessation [[15], p. 307]. Our study offers important insights into how voluntary retailer initiatives could be part of achieving these goals. First, pharmacies were not the only stores for which tobacco sales were regarded as inappropriate by owners/employees: grocery stores selling healthy products also fell into this category. For tobacco control advocates seeking to encourage retailers to voluntarily end tobacco sales, these types of stores may represent "low hanging fruit" whose successful recruitment and potential competitive advantage may eventually help denormalize tobacco sales in other grocery stores.

Recruitment efforts can emphasize potential advantages of stopping sales that we identified: making employees' jobs easier (by eliminating the need to check identification, retrieve cigarettes, and be alert for cigarette theft); creating opportunities for free positive publicity via earned media coverage; and increasing the patronage of customers who support the decision to end tobacco sales. Additional retailer incentives might include media releases from tobacco control organizations and endorsements on organization websites. Addressing any unease over limiting customers' "choices" (as expressed by a grocery store owner in our study) might be accomplished by pointing out that tobacco is not a normal grocery item, but a toxic and lethal product when used as intended. Groceries might also be encouraged to replace tobacco products with nicotine replacement products, thereby offering consistency with their vision of caring for consumers and countering the perception of limiting "choice." Alternatively, retailer resistance might best be overcome through a progressive policy that starts by first encouraging retailers to remove tobacco from open display and moves towards ending tobacco sales (an approach taken by New York state tobacco control advocates) [39,40].

Our study also showed that retailers' decisions to end tobacco sales may be at least partly precipitated by regulatory requirements, including licensing and customer identification. This suggests that enhancing or expanding such requirements may incentivize other retailers to end tobacco sales. Increasing retailer licensing fees (in California, currently a one-time payment of just $100 to the state and possibly an annual fee (typically $200-$350) [41] to the city and/or county) or creating additional licensing requirements (such as local community approval for new retail outlets during the planning submission process) should be considered. Alternatively, currently licensed retailers might be offered state or local-level incentives–such as a tax credit or cut–to voluntarily relinquish their licenses.

A surprising finding was how few customers were aware that retailers had ended tobacco sales, even in cases where businesses garnered media attention. While retailers' decision to voluntarily abandon tobacco sales disrupts the normalization of smoking created by the ubiquitous availability of cigarettes, it will be most effective only if the public is aware. Given that retailers reported minimal negative reaction among customers, a finding confirmed in focus groups, there appears to be little downside to continuously publicizing the policy. Tobacco control advocates might consider creating signage for retailers indicating that they have chosen to become tobacco-free retailers.

An intriguing possibility raised by our study is the potential link between retailers discontinuing tobacco sales and their customers trying to quit or successfully quitting smoking. Key components of this link appeared to be prior regular purchase of cigarettes at the retailers in question and customer knowledge of the policy. While the inconvenience of having to shop for cigarettes elsewhere may have motivated customers to quit or cut down, the symbolism of the decision–tobacco is a deadly product that does not belong in this store–may have also played a role.

Voluntary decisions by businesses to end tobacco sales may lay the groundwork for mandatory policies, such as limitations on the number, location, or density of tobacco retailers or bans on tobacco sales by specific categories of retailers, such as pharmacies [42]. Indeed, voluntary initiatives by pharmacies preceded recent bans on pharmacy tobacco sales in San Francisco and Richmond, California and several Massachusetts cities [43]. Our study echoes others in suggesting that banning cigarette sales in pharmacies is likely to be uncontroversial among the public and pharmacists [17,18]; banning cigarette sales in grocery stores may be the next logical step.

Conclusions

Voluntary retailer abandonment of tobacco sales both reflects and extends social norm changes that have publicly problematized tobacco in California. Our findings suggest that such voluntary initiatives by retailers are welcomed by consumers and should be incentivized, supported, and publicized, enhancing public health efforts to reduce the ubiquitous availability of tobacco in retail stores.

Acknowledgements

This work was supported by the California Tobacco-Related Disease Research Program, grant number 18XT-0140. The funder played no role in study design; in the collection, analysis, and interpretation of data; in the writing of the manuscript; or in the decision to submit the manuscript for publication. We would like to thank Vera Harrell for help with recruitment, Valerie Yerger for help with data collection, Naphtali Offen for help with data collection, coding and analysis, and Sarah Pearce for help with data coding and entry.

Authors' contributions

PAM and REM conceived and designed the study. PAM collected interview and focus group data, conducted unobtrusive observations, coded data, and wrote the first draft of the manuscript. REM reviewed interview and focus group transcripts and coded data categories and edited and revised all manuscript drafts. Both authors read and approved the final manuscript.

Competing interests

The authors declare that they have no competing interests.

Received: 12 August 2011 Accepted: 8 November 2011
Published: 8 November 2011

References

1. Cigarette and Tobacco Products Licensing and Enforcement. [http://www.boe.ca.gov/meetings/pdf/Item_P3c4__Cig_Tobacco_Prod_Lic_Enforcement_AB71_091608.pdf].
2. Novak SP, Reardon SF, Raudenbush SW, Buka SL: Retail tobacco outlet density and youth cigarette smoking: a propensity-modeling approach. *Am J Public Health* 2006, 96(4):670-676.
3. Henriksen L, Feighery EC, Schleicher NC, Cowling DW, Kline RS, Fortmann SP: Is adolescent smoking related to the density and proximity of tobacco outlets and retail cigarette advertising near schools? *Prev Med* 2008, 47(2):210-214.
4. Henriksen L, Schleicher NC, Feighery EC, Fortmann SP: A Longitudinal Study of Exposure to Retail Cigarette Advertising and Smoking Initiation. *Pediatrics* 2010, 126(2):232-238.
5. Henriksen L, Flora JA, Feighery EC, Fortmann SP: Effects on youth of exposure to retail tobacco advertising. *J Appl Soc Psychol* 2002, 32(9):1771-1789.
6. Henriksen L, Feighery EC, Wang Y, Fortmann SP: Association of retail tobacco marketing with adolescent smoking. *Am J Public Health* 2004, 94(12):2081-2083.
7. Lovato CY, Hsu HC, Sabiston CM, Hadd V, Nykiforuk CI: Tobacco Point-of-Purchase marketing in school neighbourhoods and school smoking prevalence: a descriptive study. *Can J Public Health* 2007, 98(4):265-270.
8. Slater SJ, Chaloupka FJ, Wakefield M, Johnston LD, O'Malley PM: The impact of retail cigarette marketing practices on youth smoking uptake. *Arch Pediatr Adolesc Med* 2007, 161(5):440-445.
9. Wakefield M, Germain D, Henriksen L: The effect of retail cigarette pack displays on impulse purchase. *Addiction* 2008, 103(2):322-328.
10. Paynter J, Edwards R: The impact of tobacco promotion at the point of sale: a systematic review. *Nicotine Tob Res* 2009, 11(1):25-35.
11. Hoek J, Gifford H, Pirikahu G, Thomson G, Edwards R: How do tobacco retail displays affect cessation attempts? Findings from a qualitative study. *Tob Control* 2010, 19(4):334-337.
12. Carter OB, Mills BW, Donovan RJ: The effect of retail cigarette pack displays on unplanned purchases: results from immediate postpurchase interviews. *Tob Control* 2009, 18(3):218-221.
13. Burton S, Clark L, Jackson K: The association between seeing retail displays of tobacco and tobacco smoking and purchase: findings from a diary style survey. *Addiction* , Published online, 20 July 2011.
14. Chapman S, Freeman B: Regulating the tobacco retail environment: beyond reducing sales to minors. *Tob Control* 2009, 18(6):496-501.
15. Institute of Medicine: *Ending the tobacco problem: a blueprint for the nation* Washington, DC: National Academies Press; 2007.
16. Bentley JP, Banahan BF, McCaffrey DJ, Garner DD, Smith MC: Sale of tobacco products in pharmacies: results and implications of an empirical study. *J Am Pharm Assoc* 1998, 38(6):703-709.
17. Hudmon KS, Fenlon CM, Corelli RL, Prokhorov AV, Schroeder SA: Tobacco sales in pharmacies: time to quit. *Tob Control* 2006, 15(1):35-38.
18. Hudmon KS, Hussar DA, Fenlon CM, Corelli RL: Pharmacy students' perceptions of tobacco sales in pharmacies and suggested strategies for promoting tobacco-free experiential sites. *Am J Pharm Educ* 2006, 70(4):75.
19. Kotecki JE, Torabi MR, Elanjian SI: Pharmacists' opinions and practices related to the sale of cigarettes and alcohol–a follow-up study. *J Community Health* 1997, 22(6):469-479.

20. Kotecki JE, Elanjian SI, Torabi MR, Clark JK: **Pharmacists' concerns and suggestions related to the sale of tobacco and alcohol in pharmacies.** *J Community Health* 1998, **23(5)**:359-370.
21. Kotecki JE, Fowler JB, German TC, Stephenson SL, Warnick T: **Kentucky pharmacists' opinions and practices related to the sale of cigarettes and alcohol in pharmacies.** *J Community Health* 2000, **25(4)**:343-355.
22. Kotecki JE, Hillery DL: **A survey of pharmacists' opinions and practices related to the sale of cigarettes in pharmacies-revisited.** *J Community Health* 2002, **27(5)**:321-333.
23. Parish RC, Terrell JC: **Georgia pharmacists' attitudes toward cigarettes and smokeless tobacco.** *Am Pharm* 1989, **NS29(9)**:41-46.
24. Brown LJ, DiFranza JR: **Pharmacy promotion of tobacco use among children in Massachusetts.** *Am Pharm* 1992, **NS32(5)**:45-48.
25. Eule B, Sullivan MK, Schroeder SA, Hudmon KS: **Merchandising of cigarettes in San Francisco pharmacies: 27 years later.** *Tob Control* 2004, **13(4)**:429-432.
26. Hickey LM, Farris KB, Peterson NA, Aquilino ML: **Predicting tobacco sales in community pharmacies using population demographics and pharmacy type.** *J Am Pharm Assoc* 2006, **46(3)**:385-390.
27. **Verify a license.** [http://www.pharmacy.ca.gov/online/verify_lic.shtml].
28. **We do small business.** [http://www.manta.com/].
29. Frey JH, Oishi SM: *How to conduct interviews by telephone and in person* Thousand Oaks, CA: Sage Publications; 1995.
30. Morgan DL: *Focus groups as qualitative research.* 2 edition. Thousand Oaks, CA: Sage Publications; 1997.
31. Patton MQ: *Qualitative research and evaluation methods.* 3 edition. Thousand Oaks, CA: Sage Publications; 2002.
32. QSR International: **NVivo 8.** 2008.
33. Hsieh HF, Shannon SE: **Three approaches to qualitative content analysis.** *Qual Health Res* 2005, **15(9)**:1277-1288.
34. Hammond D, Fong GT, Zanna MP, Thrasher JF, Borland R: **Tobacco denormalization and industry beliefs among smokers from four countries.** *Am J Prev Med* 2006, **31(3)**:225-232.
35. Levy DT, Hyland A, Higbee C, Remer L, Compton C: **The role of public policies in reducing smoking prevalence in California: results from the California tobacco policy simulation model.** *Health Policy* 2007, **82(2)**:167-185.
36. Pierce JP, Gilpin EA, Emery SL, White MM, Rosbrook B, Berry CC, Farkas AJ: **Has the California tobacco control program reduced smoking?** *JAMA* 1998, **280(10)**:893-899.
37. Gilpin EA, Lee L, Pierce JP: **Changes in population attitudes about where smoking should not be allowed: California versus the rest of the USA.** *Tob Control* 2004, **13(1)**:38-44.
38. **California' smoking rate reaches lowest on record.** [http://articles.latimes.com/2011/aug/06/business/la-fi-smoking-decline-20110806].
39. **Get Mad About Ads.** [http://www.smokefreecapital.org/Exposed/GetMadAboutAds.cfm].
40. Crowley CF: **Frosted doors hiding cigarettes from view.** *The Times Union. Albany* 2008.
41. **Matrix of strong local tobacco retailer licensing ordinances.** [http://www.center4tobaccopolicy.org/CTPO/_files/_file/Matrix%20of%20Strong%20Local%20Tobacco%20Retailer%20Licensing%20Ordinances%20March%202011.pdf].
42. **Reducing the availability of tobacco products at retail: policy analysis.** [http://www.nsra-adnf.ca/cms/file/Reducing_Retail_Availabilty_policy_analysis_final_2011.pdf].
43. **Local efforts.** [http://www.tobaccofreerx.org/local_efforts.html].

Pre-publication history
The pre-publication history for this paper can be accessed here:
http://www.biomedcentral.com/1471-2458/11/848/prepub

doi:10.1186/1471-2458-11-848
Cite this article as: McDaniel and Malone: **Why California retailers stop selling tobacco products, and what their customers and employees think about it when they do: case studies.** *BMC Public Health* 2011 **11**:848.

Submit your next manuscript to BioMed Central and take full advantage of:

- **Convenient online submission**
- **Thorough peer review**
- **No space constraints or color figure charges**
- **Immediate publication on acceptance**
- **Inclusion in PubMed, CAS, Scopus and Google Scholar**
- **Research which is freely available for redistribution**

Submit your manuscript at
www.biomedcentral.com/submit

BioMed Central
