# Supplementary material for: Understanding Community Norms Surrounding Tobacco Sales
Source: PLoS One. 2014 Sep 2;9(9):e106461. doi: 10.1371/journal.pone.0106461 (PMC4152285; doi:10.1371/journal.pone.0106461)
Supplement: Text S2 — New York and Ohio retailers study. (PDF) [file pone.0106461.s002.pdf]

PLOS ONE

"People over Profits": Retailers Who Voluntarily Ended Tobacco Sales

Patricia A. McDaniel*, Ruth E. Malone

Department of Social and Behavioral Sciences, School of Nursing, University of California San Francisco, San Francisco, California, United States of America

Abstract

Background: Tobacco retailers are key players in the ongoing tobacco epidemic. Tobacco outlet density is linked to a greater likelihood of youth and adult smoking and greater difficulty quitting. While public policy efforts to address the tobacco problem at the retail level have been limited, some retailers have voluntarily ended tobacco sales. A previous pilot study examined this phenomenon in California, a state with a strong tobacco program focused on denormalizing smoking and the tobacco industry. We sought to learn what motivated retailers in other states to end tobacco sales and how the public and media responded.

Methods: We conducted interviews with owners, managers, or representatives of six grocery stores in New York and Ohio that had voluntarily ended tobacco sales since 2007. We also conducted unobtrusive observations at stores and analyzed media coverage of each retailer's decision.

Results: Grocery store owners ended tobacco sales for two reasons, alone or in combination: health or ethics-related, including a desire to send a consistent health message to employees and customers, and business-related, including declining tobacco sales or poor fit with the store's image. The decision to end sales often appeared to resolve troubling contradictions between retailers' values and selling deadly products. New York retailers attributed declining sales to high state tobacco taxes. All reported largely positive customer reactions and most received media coverage. Forty-one percent of news items were letters to the editor or editorials; most (69%) supported the decision.

Conclusion: Voluntary decisions by retailers to abandon tobacco sales may lay the groundwork for mandatory policies and further denormalize tobacco. Our study also suggests that high tobacco taxes may have both direct and indirect effects on tobacco use. Highlighting the contradictions between being a responsible business and selling deadly products may support voluntary decisions by retailers to end tobacco sales.

Citation: McDaniel PA, Malone RE (2014) "People over Profits": Retailers Who Voluntarily Ended Tobacco Sales. PLoS ONE 9(1): e85751. doi:10.1371/journal.pone.0085751

Editor: Natalie Walker, The National Institute for Health Innovation, New Zealand

Received October 10, 2013; **Accepted** December 1, 2013; **Published** January 22, 2014

Copyright: © 2014 McDaniel, Malone. This is an open-access article distributed under the terms of the Creative Commons Attribution License, which permits unrestricted use, distribution, and reproduction in any medium, provided the original author and source are credited.

Funding: This project was funded by the National Cancer Institute, grant #R01 CA143076 (http://www.cancer.gov/). The funders had no role in study design, data collection and analysis, decision to publish, or preparation of the manuscript.

Competing Interests: The authors have declared that no competing interests exist.

* E-mail: patricia.mcdaniel@ucsf.edu

Introduction

Tobacco retailers perpetuate the tobacco problem [1]. Tobacco outlet density increases the likelihood of smoking among minors and adults [2–5], and living in close proximity to tobacco outlets makes quitting more difficult [6–7]. One explanation for these observed relationships is the presence of tobacco advertising and tobacco displays in tobacco retail outlets, which normalize and promote tobacco use [8–12] and trigger smoking urges among smokers and former smokers [12–13]. The ready availability of tobacco also suggests to the public that warnings about its deadliness may be overblown [14].

Particular population groups appear to be more likely to be exposed to tobacco retailers, with research noting a heavy concentration of tobacco retailers near schools, in economically and socially deprived neighborhoods, and in neighborhoods with high proportions of African Americans and Hispanics [2,15–21]. Thus, reducing the number of stores that sell tobacco might be a particularly effective means of reducing tobacco uptake and use among the most vulnerable.

In the US, there have been limited efforts to date to address the tobacco problem at the retail level, despite public support for such actions as capping the number of tobacco retailers, banning pharmacy tobacco sales, and banning tobacco sales near schools [22–24]. One hundred eleven cities and counties have banned tobacco sales in close proximity to schools [25], several towns, cities, counties, and states have raised the legal age of tobacco purchase above 18 [26–28], and two California cities and numerous Massachusetts municipalities have banned tobacco sales in pharmacies [29]. Wider application of these policies could have a significant impact; for example, a statewide pharmacy tobacco sales ban would eliminate nearly 10% of tobacco retailers in Massachusetts [30].

Despite the absence of a strong public policy response in most jurisdictions, however, some retailers have voluntarily enacted stricter policies around tobacco sales [31], including ending tobacco sales altogether. In a pilot study, we explored why some

California grocery stores and pharmacies had done so [32]. We found that retailers frequently cited tobacco's negative health effects as an impetus to discontinue tobacco sales. However, given California's strong tobacco control program, which has focused on denormalizing both smoking and the tobacco industry [33], California retailers may be uniquely motivated to voluntarily abandon tobacco sales. Therefore, we sought in a larger project to explore the phenomenon in other states. This paper examines grocery stores in two states outside California that have discontinued tobacco sales. Using interviews, observations, and an analysis of media coverage, we explored their characteristics, tobacco retail environments, reasons for ending tobacco sales, and public opinion in order to understand their similarities to and differences from their California counterparts.

Methods

Ethics Statement

The study was approved by UCSF's Committee on Human Research (CHR), IRB #10-00850. We agreed not to reveal in publications the names of the businesses or anyone we interviewed. In order to further protect businesses' identities, the interview data are not available to the public.

Our goal was to identify and recruit any grocery store outside of California that had voluntarily ended tobacco sales since 2007 (within five years of data collection, in order to ensure adequate recall). Because many states do not have tobacco retail licensure, we could not use such data to identify those relinquishing such licenses. Instead, we searched the Lexis Nexis, Proquest, and Access World News online databases for items referencing such retailer actions. Although not every retailer publicizes the decision to end tobacco sales, our initial searches of the media databases suggested that relying on this method would yield a substantial number of eligible businesses. The three databases covered 1,441 news sources, including 995 local and national newspapers, 11 magazines, 60 newswires, 256 web-only news sources, 26 ABC, CBS, NBC, and Fox news broadcasts, and National Public Radio news broadcasts. We used a variety of search terms intended to capture news items concerning all supermarkets and grocery stores who had voluntarily ended tobacco sales (e.g., (grocer* or chain or retailer or supermarket) AND (stop or end or drop or quit or eliminat* or remov* or discontinue) AND (tobacco or cigarette or smok*)).

We found 11 groceries in New York, New Jersey, Michigan, and Ohio that fit our criteria. After being contacted by phone by the first author and informed of the nature of the study and the reasons for doing the research, six agreed to participate, five in New York and one in Ohio. Two declined to participate, two failed to respond to interview requests over a two-month period, and one grocery owner who had agreed to an in-person interview did not appear. Non-participating grocery stores were similar to participating groceries in terms of size, product types, and median household income rank of neighboring communities.

For each retailer, the first author obtained and made written note of oral informed consent (as approved by the CHR) and conducted a 20–30 minute in-person (n = 3) or telephone interview (n = 4) with an owner or store manager; these individuals were typically most involved in creating and/or implementing the voluntary tobacco-related policy (table 1). In the case of the largest grocery store chain, the first author spoke to the consumer relations director, rather than the owner or an individual store manager. (She had also been the consumer relations director when the store went tobacco-free, and was involved in discussions about ending tobacco sales.) Interview questions were pilot tested in the California retailer study. They explored why and how the tobacco-free policy was created, implemented, and advertised, its financial impact, customer and community reaction, and interviewees' satisfaction with the policy. All interviews were audiotaped. Face-to-face interviews were conducted in private offices, with no one else present; interviews conducted by phone gave the impression of taking place in a private room when the interviewee was alone. There were no discernible differences in quality between face-to-face and telephone interviews. There were also no discernible differences in interviewees' ability to recall events according to how much time had elapsed since making the decision to end tobacco sales; while some elaborated more than others, this appeared to be more a matter of personality than memory. We refer to the businesses by state abbreviation (NY or OH) and an assigned number (e.g., "NY grocery 1"). We refer to interviewees by job title.

Interview transcripts were transcribed by professional transcribers and checked for accuracy by the first author. The first author coded the transcripts using a codebook created for the pilot project. In that project, four coders, including both authors, created a codebook through a collaborative, inductive process involving data review and discussion of key points [32]. We created an initial set of codes collectively; as data review progressed, we refined and added codes, re-coding earlier transcripts to reflect changes. For this project, we used the software package NVivo9 for data management [34]. Given our interest in providing in-depth knowledge of retailers' decision to end tobacco sales, we analyzed interview data using qualitative content analysis, which involves identifying themes or patterns in systematically coded text [35]. We chose quotes that were representative of the themes we identified.

The first author also conducted unobtrusive observations at each store (for chain stores, we randomly selected one store in the chain), guided by an observation inventory created by both authors and pilot tested and refined during the California retailer study. The inventory encompassed store characteristics, such as size and location; product types, including whether the store sold nicotine replacement therapy; signs advertising the tobacco-free policy or other health messages; items for sale at the customer service counter; and the number of tobacco retailers within a three-block radius. In cases where the store owner had agreed to an in-person interview, the first author conducted the observation several hours before the interview was scheduled. In all but the smallest stores, she spent at least 30 minutes inside the store, filling out the inventory as unobtrusively as possible. In smaller stores, where lingering while filling out a form might have raised suspicions, she spent less time in the store and filled out the inventory immediately after leaving.

We returned to the three online media databases (Lexis Nexis, Proquest, and Access World News) to capture ALL news items concerning the retailers in our study, using search terms similar to those outlined above but also including retailers' names. Four coders, including both authors, coded news items through a collaborative, multi-step process, coding story characteristics (i.e., news source, story type, date, etc.) and content (additional details are available elsewhere) [36]). For the purposes of this paper, we focused our analysis on volume of coverage, story type, and support (or not) for the voluntary policy expressed in editorials or op-eds and letters to the editor.

Finally, we gathered information on New York and Ohio's tobacco retail environments from government and tobacco control websites, and spoke to a New York tobacco control organization to obtain further details about its retailer-focused media campaign.

Table 1. Participating grocery stores and pharmacies.

Name	Number of stores	Year tobacco sales ended	Median household income rank of neighboring community (0–99)*	Number of tobacco retailers within 3-block radius of (selected) store	Interviewees (N = 7)	Number of news items published about retailer
NY Grocery 1	83	2008	5–98	2	Consumer relations director	155
NY Grocery 2	3	2008	62–63	3	Owner; manager	11
NY Grocery 3	1	2009	71	1	Owner	1
NY Grocery 4	8	2008	93–99	2	Owner	19
NY Grocery 5	1	2007	99	3	Owner	3
OH Grocery 1	3	2008	78–95	3	Manager	4

*From http://zipwho.com.
doi:10.1371/journal.pone.0085751.t001

Triangulation of data (interviews, observations, media analysis, and website information) provided cross-data validity checks [37].

Results

Tobacco Retail Environments

New York increased the state cigarette tax by $1.25 to $2.75 per pack in 2008, and to $4.35 per pack in 2010 [38], making it the highest state tax in the United States [39]. New York requires retailers to purchase a license to sell tobacco and to pay an annual licensing fee; localities within the state may impose additional licensing requirements and fees. In 2009, the state raised the fee from $100 to $1000–$5000, depending on sales volume, in order to reduce the number of tobacco retailers and close a budget gap [40]. The fee hike was challenged in court by retail trade associations, and in 2011 the state legislature set the fee at $300 [41–42].

In 2007, a New York tobacco control coalition initiated a media campaign to encourage local tobacco retailers to voluntarily remove or reduce tobacco advertising in stores. When the largest retailer in our study ended tobacco sales, the coalition held a media event, and took out newspaper and radio advertisements to draw attention to the store's decision. It also supported the New York State Department of Health's 2008 advertising campaign urging grocery stores and pharmacies to stop selling tobacco products (figure 1) by helping with the cost of ads and printing post cards to be handed out at informational events.

In Ohio, the state cigarette tax is $1.25, last raised in 2005 (from $0.70) [38]; it is lower than the current US median cigarette tax of $1.36 (as of 1/1/13). Like New York, Ohio requires retailers to purchase an annual tobacco license, for $125; prior to 2010, the annual fee was $25 [43]. Ohio tobacco control organizations have not implemented any retailer focused campaigns similar to those in New York.

Description of Grocery Stores

The grocery stores in our study varied in size, and included a deli that sold a handful of groceries, an independent grocery store, three small to medium-sized grocery chains with 3–8 stores, and one large 83-store regional grocery store/pharmacy chain (based in New York but with 45% of its stores spread among five other Northeastern states) (table 1). (All of the chains were technically supermarkets because they sold household items as well as food, but for simplicity's sake we use the term grocery store to refer to all retailers that sold groceries.) All of the grocery stores were privately owned; four were family-run businesses with two generations currently or formerly occupying leadership positions.

NY grocery 4 and OH grocery 1 were high-end, selling specialty health products (e.g., organic produce, gluten-free foods, and "natural" personal care products) and located exclusively in relatively affluent communities (table 1). In addition to grocery items, deli and seafood counters, and a bakery, the OH grocery 1 location visited by the first author contained sushi, salad, cheese, and wine bars, a pizza oven, and a cooking school. Similarly, the NY grocery 4 store the first author observed contained a pizza restaurant and sushi bar in addition to more standard bakery, seafood, and meat counters.

NY grocery 1 offered a wide variety of products for all budgets, including specialty health products, with stores located in economically disadvantaged as well as affluent communities (table 1). One location contained a marketplace offering a variety of prepared foods and drinks, including pizza, sushi, sandwiches, juices, and coffee, but a large section of the store was also devoted to lower-priced, family-sized frozen and fresh food. It also had a pharmacy. NY groceries 2 and 3 appeared to cater to middle income customers, as they did not offer expensive specialty products and were located in middle-income neighborhoods. While both stores had meat counters and bakeries, and NY grocery 2 had a café selling pizza, chicken, and fish, they did not offer the wide range of in-store prepared foods available at the two high end stores. NY grocery 5 was a small deli located in an affluent area; however, its product offerings – coffee, sandwiches, candy, chips, fruit, salads – were accessible to a variety of budgets.

All five of the New York groceries sold beer, the only type of alcohol (other than wine coolers) legally allowed for sale in groceries in New York state [44]. The Ohio grocery store sold beer, wine, and some liquor, as permitted by state law [45]. There were 1–3 tobacco retailers within a three-block radius of each store observed (table 1). When still selling tobacco products, none of these stores reportedly had displayed tobacco advertisements (other than the cigarette packs themselves which, in all but the deli, were displayed and sold at customer service counters). However, most (4) participated in tobacco industry incentive programs for preferential display of particular brands. Tables 2–4 offer more detailed descriptions of a sampling of the stores.

Making the Decision to End Tobacco Sales

Grocery store owners offered two reasons for ending tobacco sales, alone or in combination: health/ethics-related, or business-related. Health-related reasons were given by four groceries. For example, the owner of NY grocery 5 was a Christian who always

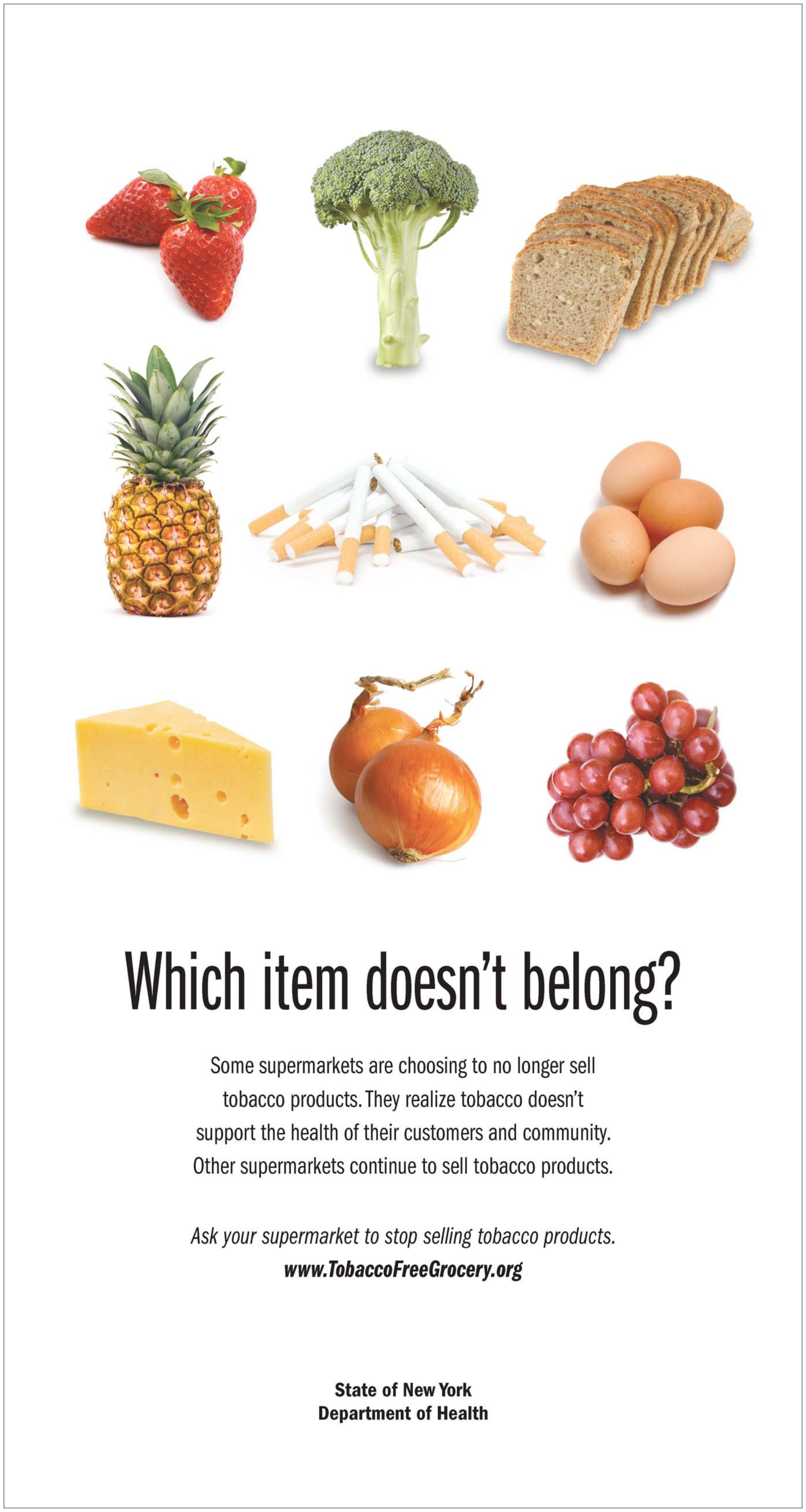

Figure 1. New York State Department of Health advertisement.
doi:10.1371/journal.pone.0085751.g001

Table 2. Detailed description of New York grocery 1.

This 83-store regional supermarket chain is based in western New York, but has 45% of its stores spread among five other Northeastern states. It is a family-owned business founded in the early 20th century, with 42,000 employees. One of the stores visited by the first author was a 24-hour, 140,000 square foot "super store" that occupied an entire strip mall in a city of nearly 260,000. The store had 22 aisles of grocery and non-grocery items (e.g., greeting cards, cosmetics, personal care products); a produce section; meat, seafood, and cheese counters; a bakery section (both in-store and commercial, and a French "patisserie"); a frozen food section; a "Nature's Marketplace" stocking organic, "natural," and vegetarian-friendly foods; a pharmacy; a catering service; and a prepared and made-to-order food marketplace offering sushi, chicken, pizza, sandwiches, and Chinese and Thai food. NY grocery 1 also offered free wireless internet access and a "fun center" where parents of children aged 3–8 could drop off their children while they shopped. The store was notable for selling, in the words of the consumer relations director, "soup to caviar. … We have the ordinary and the extraordinary." NY grocery 1 also sells its own brand of various grocery items, such as peanut butter, bread, and organic produce. The customer service counter, where cigarettes were formerly sold, was close to the store entrance. It sold lottery tickets and postage stamps, and offered check cashing and rug cleaning services. The pharmacy, in the middle of the store, sold nicotine replacement therapy products. The pharmacy had a display of "books for your health" (none of the titles explicitly mentioned tobacco); there were also several other health messages displayed on banners in the produce and bakery sections, noting the healthfulness of organic crops and of whole grains. The family that owns NY grocery 1 were reportedly "never fond" of selling cigarettes "because of all the health concerns associated with smoking," but did so because they thought customers appreciated the convenience. In 2007, the CEO began discussing with management and the consumer affairs department the idea of ending tobacco sales. In addition to health concerns, the decision was prompted by a desire for consistency with the company's plan to offer employees and their spouses a free smoking cessation program and with its healthy eating and living messages to employees and customers. In discussions about the proposed policy, it came up that "we sell a lot of things that aren't necessarily good for you. … We sell beer. In some states we sell wine and liquor. We sell chocolate cake and chocolate candy. But in the end we said, 'But all of those things are good in moderation. Smoking, there's no acceptable level or frequency for smoking'" (Consumer relations director, NY Grocery 1). When the owner made the decision to end tobacco sales, the store first sent employees a letter, and then issued a press release and put up signs informing customers that in 5 weeks the store would no longer sell tobacco products.

doi:10.1371/journal.pone.0085751.t002

felt conflicted about selling tobacco products. However, his business partner was a smoker, so "it would have been very difficult to stop while we were partners." But once he became sole owner, he said, "How can I justify this?":

> I'm a Christian. … I talk to people about being responsible in terms of things they do and don't do. And yet I was making money on selling cigarettes. I'm a local fire chief. You know, smoking materials is like the number one cause of fatal residential fires. … And I had a brother-in-law who passed away from lung cancer. He was a smoker. … So you know, kind of putting all those things together [led to my decision]. (Owner, NY grocery 5)

The ambivalence captured in this statement reflects the owner's multiple roles–as religious person, community member and fire chief, and family member–and his effort to reconcile responsibly carrying out these roles with his role as a businessman selling harmful products. This suggests that the increasing public awareness about the multiple ways tobacco causes harm may help make it more challenging for owners to justify profiting from its sale. For some owners already experiencing such ambivalence, local community pressure could help facilitate a decision to discontinue sales.

Similarly, NY grocery 1 cited ethical contradictions between its healthy eating and activity messages to employees and customers, and selling tobacco products. In addition, the store planned to offer employees and their spouses free smoking cessation services and felt that it could not do so and still sell tobacco: "It's kind of hard to introduce a program like that and still sell tobacco. That … was the … challenge that we were faced with. … How do you talk to employees about this kind of thing and sell tobacco?" (Consumer relations director, NY grocery 1). As with the owner above, the decision was made to resolve troubling contradictions between selling tobacco and being a responsible employer.

Business-related reasons for ending tobacco sales (given by four groceries) consisted of declining tobacco sales or a poor fit with the store's image. The New York groceries attributed high tobacco taxes to declining sales. As NY grocery 2's owner explained:

> We have around here a lot of … Native American … [reservations], where they're selling cigarettes without the taxes on it. So sales of cigarettes … the last … 20 years have been decreasing … because there's been more people going to the reservations to buy the cigarettes. So, … we …looked at it like, "Well, you know, we're not selling the kind of cigarettes we were years ago, and it would be a good thing to do it."

Table 3. Detailed description of New York grocery 2.

This 3-store grocery store chain with 500 employees is located in western New York. It is a family-owned business, in operation for over 80 years. The current owner has been involved in the store for over 50 years. One of the stores visited by the first author was situated in a strip mall in a city of nearly 32,000 residents. A dry cleaner, independent pharmacy, and hair salon were located in the same strip mall, and three tobacco retailers were nearby. The store itself is approximately 38,000 square feet, and has 15 aisles, in addition to a large produce section, meat counter, deli, bakery, and café selling pizza, chicken, and sandwiches. The store also sold some store-branded items (e.g., canned food). There were no nutrition or other health-related messages on display in the store. Upon entering the store, customers passed the customer service counter, where cigarettes were formerly sold. It offered gift cards, stamps, and lottery tickets; at one time, nicotine replacement therapy products were sold there. There was a picture of the owner and his son near the entrance of the store; when the first author visited, the owner was walking the aisles of the store, interacting with customers and employees. The owner, his son, and two managers collectively made the decision to end tobacco sales in 2008. For several years, they had discussed ending tobacco sales, but did so only after NY grocery 1 "gave them the green light" by also ending sales. The owner explained that they were motivated to end sales by health and business reasons. He didn't elaborate on specific health concerns, except to say that "it's not good for people to be smoking." He also expressed a desire to set a good example for his younger employees, many of whom smoked, although none claimed to have stopped smoking due to the decision. Steadily declining tobacco sales played a big part in the decision as well. At one time NY grocery 2 had sold $20,000 worth of cigarettes per week; by the time the store ended sales, that figure had dwindled to $200- $1,000 per week. The store gave customers 3-weeks' notice that tobacco sales would be ending, and continued to sell remaining inventory during that time. On the appointed end date, all remaining cigarettes were reportedly thrown "in the dumpster."

doi:10.1371/journal.pone.0085751.t003

Table 4. Detailed description of Ohio grocery 1.

Ohio grocery 1 is a three-store self-described ''gourmet supermarket'' with 700 employees, headquartered in Dayton, Ohio. It was founded in the late 1940s, and continues to be owned and operated by one of the founder's families. The store visited by the first author was located in a strip mall in a suburb of Dayton with a population of nearly 24,000. It had 12 aisles of food and personal care products; seafood and meat counters; a deli; produce section; bakery; pizza oven; salad, cheese, and wine bars; a florist; and a cooking school next door. The store regularly hosts food and wine tastings, and sells a wide variety of organic, gluten free, and specialty foods. It does not sell nicotine replacement therapy products. The store did not have any health-related messages on display, and did not advertise its tobacco-free policy in the store; however, an exterior sign advised that there was ''no smoking inside the store.'' In 2013, one of the stores hosted a ''health fair'' featuring informational materials on healthy foods and activities as well as samples of various healthy food products. Tobacco products were formerly sold in two of the three stores; when the newest store opened in 2002, it did so without tobacco sales. They received little negative feedback for failing to stock tobacco. Six years later, tobacco products were removed from the remaining two stores. One sold sushi in the space formerly occupied by tobacco products, while the other sold chocolates. The father and son owners of the store made the decision to end tobacco sales, and the managers supported it because ''we all agreed … that we could probably do more with that space … than the cigarettes were doing with it'' (Manager, OH grocery 1). The manager asserted that tobacco's negative health effects was not a motivation for ending sales. Instead, the decision was a straightforward business calculation: tobacco sales were ''a dying category'' among their customers and tobacco did not fit with the store's focus on ''selling great food'' (Manager, OH grocery 1). Customers were informed of the impending change by word-of-mouth, and, while some were disappointed at the inconvenience, most of them understood the decision. OH grocery 1 did not advertise its decision publicly, but it did inform members of an informal group of specialty retailers to which it belonged that it had stopped selling tobacco and that there had been no negative impact.

doi:10.1371/journal.pone.0085751.t004

Even here, however, the focus on abandoning sales being a "good thing to do" suggests an underlying ethical motive in addition to the declining sales. The Ohio grocery chain attributed its declining tobacco sales to its customer base, higher income individuals who tend to be non-smokers. The store also concluded that selling cigarettes did not fit with its image as a source of great food: "We were all about selling great food, and we thought we could use the efforts and energy and spaces … designated for tobacco use to sell what we believed in. … So we got out of it" (Manger, OH grocery 1).

The idea to end tobacco sales was initiated by the owners, rather than managers, employees, customers, or tobacco control organizations. However, two retailers acknowledged that NY grocery 1's decision stimulated additional interest on their part in the idea to end tobacco sales, because it was "the industry leader."

Implementing the New Policy

All but one of the grocery stores phased in the new tobacco-free policy gradually, discontinuing tobacco orders and selling existing stock, sometimes at a discount. Four retailers posted signs to alert customers to it; NY Grocery 5 (the deli), and OH Grocery 1 did not. Typically, the signs did not offer a justification or reason for the change, but simply stated, according to the owners, that "as of such-and-such [date] we're no longer going to be selling cigarettes" (Owner, NY grocer 2). However, NY grocery 1, the largest retailer in our study, and one for whom the negative health effects of tobacco were the sole motivation for ending tobacco sales, explained on its sign that "We have come to this decision after thinking about the role smoking plays in people's health" (Consumer relations director, NY grocery 1). Management was concerned about alienating customers and "wanted to make it clear … we weren't trying to be judgmental here. We were just trying to do what we thought was the right thing" (Consumer relations director, NY grocery 1). Before announcing the decision publicly, the owner sent a letter to employees explaining it and informing smoking employees that they and their spouses could participate in a new, free smoking cessation program.

Fearing a loss of customers, the smallest retailer in our study chose not to post a sign. Instead, he handed out letters to regular cigarette buyers explaining the difficulty of the decision and asking them to continue patronizing the store:

> Dear Valued Customer, after months and months or years of thinking, [NY grocery 5] has made the difficult decision to stop selling cigarettes, tobacco products, cigars, etcetera, effective immediately, based on health concerns, loved ones, our principles. However, we would love our valuable customers to continue coming for the good food and the good drink and the good service. (Owner, NY grocery 5)

The owner also took some customers aside and said, "Listen. I hope you keep coming, and I hope you understand my reasons. I'm not forcing my morals on anybody" (Owner, NY grocery 5). Rather, the decision was positioned as resolving the owner's own internal contradictions between his "principles" and continuing to sell harmful products.

After a certain period, retailers who had posted signs removed them and did not advertise their tobacco-free status in stores or on websites. Their assumption was that "everyone knows" that the store no longer sold cigarettes, in part because so few customers asked for them; thus, a sign was superfluous.

Publicizing the New Policy

Several tobacco control organizations in New York helped publicize the New York retailers' decision to end tobacco sales by publishing ads in local newspapers (including full page and color ads) thanking the stores for their courage in "putting … customers' well-being before … profits" or proclaiming the owners' status as "tobacco free champions" and encouraging other businesses to follow their example. One organization released an ad asking when other New York supermarkets would follow the lead of three of the retailers in our study and "kick butts" out of their stores. By contrast, tobacco control organizations in Ohio did not help publicize the tobacco-free status of the Ohio retailer in our study.

Most retailers also received some media coverage when they ended tobacco sales, further publicizing the new policy (table 1). NY grocery 1, the only retailer to issue a press release, received the most coverage (155 news items); its large size may have also played a role in generating attention. For all retailers, most of the coverage (74%) consisted of local newspaper articles, although NY groceries 1, 2, and 4 received coverage in national newspapers such as the *New York Times* and *USA Today,* and NY grocery 1 was featured in one NBC nightly news broadcast.

Public Opinion

Forty-one percent of news items were letters to the editor (65) or editorials (15), with all but three concerning NY grocery 1. Among the letters to the editor, 69% (45) supported the retailers' decision to end tobacco sales; just 27% (12) of these supportive letters were written by regional or local tobacco control advocates or health

professionals, departments, or organizations, such as the American Lung Association of the Mid-Atlantic. One Pennsylvania customer wrote: "What a breath of fresh air it was to see the good news on the front page … about [NY grocery 1] stopping the sale of tobacco products! I applaud the management. … I will be even more pleased to support them now" [46]. Similarly, 80% (12) of the editorials praised the retailers' decision. For example, a local New York state newspaper noted that "[NY grocery 1′s] decision rightly drew praise from anti-smoking advocates, and we second that. By all accounts, this appears to be a non-stereotypical corporate move to put people over profits, and it's good for public health" [47]. The handful of editorials opposed to the decision asserted that ending tobacco sales would not help smokers quit smoking [48–49], or questioned NY grocery 1's stated concern for customers' health, noting that it sought to sell alcohol at its stores [50].

In New York, tobacco control and/or public health organizations also honored retailers who had voluntarily ended tobacco sales with awards and commendations. For example, Action on Smoking and Health sent NY grocery 5's owner a thank you letter and a $1,000 donation "with the hope that it [would] make a small difference" in the retailer's profitability. The owner also received a plaque at a "No Thanks, Big Tobacco" annual appreciation dinner sponsored by a group of regional tobacco control organizations.

Perceived Customer Reaction and Financial Impact

The retailers who based their decision to end tobacco sales solely or partly on health/ethics anticipated more negative customer reaction than retailers who were motivated solely by business-related reasons. For example, NY grocery 1 was "pretty sure that we were going to get a lot of negative reaction from customers who … were upset that we [were] … taking the convenience away from them" (Consumer relations director, NY grocery 1). However, *all* retailers reported largely positive reactions, regardless of motivation, size, or the means by which they informed customers of their decision to end tobacco sales. There were typically some complaints from smokers (and the occasional nonsmoker who objected on principle to a retailer limiting "choice"), but these "paled in comparison" to the compliments (Consumer relations director, NY grocery 1). The smallest retailer, who had the most to lose from a negative customer reaction and who adopted a highly personalized approach to informing smoking customers via letter of his decision, reported that the response was "overwhelmingly positive" even among some smokers, who said "it's inconvenient, but I respect what you're doing" (Owner, NY grocery 5). Two other retailers also reported positive feedback from smokers, in one case because they thought the retailers' decision to end tobacco sales might help them quit smoking due to the inconvenience of having to shop elsewhere for cigarettes. (NY grocery 5's owner reported that one customer did, in fact, quit smoking when the deli stopped selling cigarettes, and said it was for that very reason.).

The impact on customer loyalty of retailers' decision to end tobacco sales was more varied than the largely positive customer feedback might have suggested. Three retailers reported no customer gains or losses, while one noticed a temporary loss of customers. Two retailers assumed that they lost customers for whom cigarettes were an important draw. The deli owner explained his gut feelings about the extent of his losses:

> Now what I couldn't quantify is what I term "collateral damage." How many customers would stop coming into [my deli] because they couldn't buy a roast beef wedge, and a soda, and a bag of chips, and their pack of Marlboro? And those are the individuals that I really hated to lose. … So the collateral damage, how much additional business did I lose? I figure probably about three percent. You know, I don't have a computer model to show you how I arrived at that, but that's just my gut. (Owner, NY grocery 5)

Regardless of whether retailers reported a loss of customers, most reported a loss in sales due to the elimination of tobacco from store shelves. For most, the loss was either not substantial (as would be expected, given that declining sales prompted some retailers to end tobacco sales) or, if it was substantial, did not affect overall profits. However, the smallest and largest groceries in our study reported a considerable loss in profits. Their size may have played a role; they were also among the few businesses in our study that did not cite declining tobacco sales as a motivation to stop selling tobacco. NY grocery 1 stated that "it was $1 million to our bottom line" (across 83 stores); nonetheless, the owners reportedly did not regret the decision: "I guess we thought about it as the intelligent loss of business. You know, you're doing the right thing and living with the consequences … regardless of … where it lands" (Consumer relations director, NY grocery 1). The owner of NY grocery 5 stated that that "I know that the day I stopped selling cigarettes, I took a $50,000 a year cut in salary. And I don't make … And that's not $50,000 out of a million" (NY grocery 5, owner). Nonetheless, he, too, did not regret the decision, stating:

> I was willing to do it. Hey, it would be like you working and making … $200,000 a year, and you liked your job, but your boss said, "Listen. As part of your job, from now on you're going to have to maybe kill a person every once in a while." … And you say, "You know what? I love my job, but it's just I don't feel comfortable doing that, so I'm going to quit that job, and I'm going to get a job and make less."

Thus, even for retailers who claimed to have lost profits, the decision was regarded as reconciling their values with their actions.

Management Satisfaction with the Policy

Everyone we spoke with was satisfied with the policy of not selling tobacco products, and most could not imagine any conditions under which the policy would be altered or rescinded. All but one interviewee also stated that they wouldn't change anything if they had the chance to do it over again; that interviewee would have stopped selling cigarettes sooner. However, for three interviewees, their satisfaction with the policy was tied to its financially neutral impact. If the decision were to involve a financial cost, these interviewees' support could vanish. A NY grocery 2 manager thought that if tobacco companies gave the store "an awesome, awesome deal to sell cigarettes," it should sell them again. The owner of the store acknowledged that if he had noticed a steep decline in sales following the removal of cigarettes, "we probably would have rescinded it." The OH grocery manager thought that the decision to end tobacco sales at his store was "easy" given its largely non-smoking clientele; however, he stated that "if it's a growing category for you, I don't think I would recommend discontinuing it."

Retailers also noted several advantages (both expected and unexpected) of their decision, including improved cash flow (due to less inventory sitting on shelves), the elimination of the problem of stolen cigarettes (usually by employees), positive media attention, and an improved public image. For two retailers, the media and

community attention was surprising. NY grocery 1 expected some local media coverage, but not national media coverage or accolades and awards from public health groups and state and local governments. The owner of NY grocery 2 was surprised that his store was featured in the local newspaper and when a local tobacco control coalition "wanted to take my picture and put it on an advertisement about us not selling cigarettes. … It was a $20,000 ad in Sunday's paper, in color. That was awesome."

Attitudes towards Mandatory Policies Governing Tobacco Sales

We asked interviewees their opinion of mandatory policies governing where tobacco should be sold, using as an example San Francisco's 2008 law banning tobacco sales in pharmacies. Interviewees were nearly evenly divided on the idea, with four supporting it and three opposed. Those who opposed it did so because they thought "every retailer should have that opportunity to decide" (Consumer relations director, NY grocery 1) whether or not to sell tobacco, which was a "legal" product (Manager, OH grocery 1). Those who supported a mandatory policy did so for a variety of reasons. Two interviewees thought that limiting tobacco outlets through legislation would reduce smoking prevalence. As a manager explained, "if [smokers] have to go and search for something, chances are they're not going to want to buy it. … The harder you make it for them, … they're going to change their mind, [asking themselves] 'Do I really need that cigarette?'" (Manager, NY grocery 2). Several retailers supported a law as a way to protect children. The smallest retailer in our study, and one who suffered financially for his decision to end tobacco sales, also expressed support for a law that prohibited tobacco sales where food was sold because it would allow him to compete on "an even playing field" (Owner, NY grocery 5).

Limitations

Our study has several limitations. In the absence of universal tobacco retailer licensure, there is currently no feasible way to identify all grocery stores that have ended tobacco sales voluntarily; thus, our findings cannot be generalized to all such stores. Its geographical focus was also limited to two states, one with a high tobacco tax; retailers in other states, particularly those without high tobacco taxes, may have different motivations for ending sales. In addition, our affiliation with a health sciences university may have resulted in a response bias among interviewees, leading them to over-emphasize the role of health in their decision to end tobacco sales. (However, this appears unlikely, given the close congruence between media accounts of retailers' reasons for ending tobacco sales and our interviewees' stated reasons.).

Discussion

Retailers appeared to have resolved emerging contradictions in making the decision to stop selling tobacco. These included contradictions in their own religious, community and business values that were increasingly at odds with profiting from harmful tobacco sales, as well as the contradiction of promoting employee health while doing so. This suggests that efforts to further highlight these contradictions might be fruitful in encouraging other retailers to consider ending sales.

New York and Ohio retailers offered explanations similar to those of their California counterparts in accounting for their decision to voluntarily end tobacco sales, citing health concerns and declining tobacco sales [32]. However, in New York, unlike in the other two states, high tobacco taxes were an important explanation for declining tobacco sales. This suggests that raising tobacco taxes not only reduces smoking prevalence directly [51], by reducing consumption, but may also do so indirectly, by reducing the number of tobacco outlets. Reducing the number of tobacco outlets decreases the likelihood of smoking initiation [2–3], enhances smoking cessation [6–7], and further denormalizes smoking [14] and tobacco sales. Retailers who compete with one another may also speed up the denormalization process, as we saw in New York, where several grocery stores ended tobacco sales soon after NY grocery 1, their main competitor, did so. Indeed, New York's retailer-focused advocacy campaign relied to some extent on this dynamic to encourage other retailers to end tobacco sales.

New York's high tobacco taxes may also help explain why, unlike California and Ohio groceries, New York groceries that voluntarily ended tobacco sales were not exclusively high end. New York's higher tobacco taxes discouraged smokers from purchasing tobacco at all but the cheapest locations; thus, even grocery stores that were not upscale saw declines in tobacco sales, facilitating retailers' decision to stop selling. Grocery store owners in states without high tobacco taxes who cater to a diverse clientele may not be experiencing significant declines in tobacco sales. Advocates seeking to encourage these retailers to end tobacco sales may therefore face a more challenging task. Focusing efforts on substantially raising the tobacco licensing fee (as New York tried to do) or offering tax incentives to give up a tobacco license may prove to be the most effective strategies in such states.

New York was also unique because local tobacco control advocacy groups had an organized campaign to encourage retailers to voluntarily end tobacco sales and to publicly thank those who had done so. No retailers we spoke to mentioned this campaign as influencing their decision; however, the organizations' post-decision media efforts (including letters to the editor and full-page color newspaper "thank you" advertisements) likely drew further positive attention to the retailers and may have established a precedent for viewing such decisions as praiseworthy, appropriate, and worth emulating rather than as one-off, extreme reactions. The California Tobacco Control Program's recent initiative focused on changing the tobacco retail environment [52] could consider a similar approach. California and other jurisdictions might also consider expanding the New York initiatives' focus on the impact of tobacco sales on customers (figure 1) to include employees, as our results showed that concerns about both employee and customer health influenced some retailers' decision making.

Our study highlights additional media strategies for advocates promoting a voluntary end to tobacco sales among retailers. One is to encourage retailers to issue a press release to enhance the potential for earned media coverage, which, our study suggests, is likely to be positive. NY grocery 1, the only retailer to issue a press release, received extensive positive media attention; although its large size likely contributed to media interest, the press release may have stimulated it. (Similarly, in our study of California retailers, grocery store owners who contacted local media received the most media coverage [32].) A second strategy is to write supportive letters to the editor. While a substantial portion of media coverage consisted of letters to the editor, a relatively small number were written by tobacco control advocates or public health organizations. Such letters demonstrate additional public support to retailers, counter any negative editorials or letters, and help advertise these decisions and their positive implications for public health.

Advocates may also consider adopting strategies to discourage policy reversals. Several interviewees noted that their support for

the policy was contingent on its continued negligible financial impact, suggesting that some retailers might be vulnerable to financial incentives offered by the tobacco industry. To reinforce retailers' commitment to the policy and their self-image as tobacco-free retailers, local schools or community groups whose members benefit from the policy could be enlisted to thank retailers for ending tobacco sales (via letters or award ceremonies), endorse patronage of the businesses by community members, and regularly inform the retailer of the policy's ongoing benefits to the community (e.g., the cumulative number of tobacco point-of-sale ads avoided). Advocates may also consider combining support for existing tobacco-free retailers with initiatives focused on encouraging additional retailers to abandon tobacco sales. Such campaigns could include a positive ''these retailers support our community by not selling deadly tobacco'' message for the general public with messages that call attention to the multiple ways tobacco hurts communities, including some of those mentioned by retailers in this study, such as fires.

Voluntary decisions by retailers to end tobacco sales may lay the groundwork for mandatory policies. In San Francisco, for example, the majority of independent pharmacies had voluntarily abandoned tobacco sales long before the law formally prohibiting such sales was passed [53]. We found some support among retailers in our study for legislation barring tobacco sales in grocery stores; this was surprising, given the powerful role of the ''freedom of choice'' and ''slippery slope'' arguments in public debate about tobacco [54–55]. Recognition voiced by several interviewees that further reducing the number of outlets would reduce smoking prevalence was also encouraging. These findings suggest that tobacco control advocates may find some support in the business community in a move towards tobacco ''endgame'' ideas focused on broader restrictions on tobacco sales (including possibly phasing out at least some types of tobacco sales altogether) [14,56–60].

Author Contributions

Conceived and designed the experiments: PAM REM. Performed the experiments: PAM REM. Analyzed the data: PAM REM. Contributed reagents/materials/analysis tools: PAM REM. Wrote the paper: PAM REM.

References

1. Institute of Medicine (2007) Ending the tobacco problem: a blueprint for the nation. Washington, DC: National Academies Press.
2. Novak SP, Reardon SF, Raudenbush SW, Buka SL (2006) Retail tobacco outlet density and youth cigarette smoking: a propensity-modeling approach. Am J Public Health 96(4): 670–676.
3. Henriksen L, Feighery EC, Schleicher NC, Cowling DW, Kline RS, et al. (2008) Is adolescent smoking related to the density and proximity of tobacco outlets and retail cigarette advertising near schools? Prev Med 47(2): 210–214.
4. Lipperman-Kreda S, Grube JW, Friend KB (2012) Local tobacco policy and tobacco outlet density: associations with youth smoking. J Adolesc Health 50(6): 547–552.
5. Adams ML, Jason LA, Pokorny S, Hunt Y (2013) Exploration of the link between tobacco retailers in school neighborhoods and student smoking. J Sch Health 83(2): 112–118.
6. Reitzel LR, Cromley EK, Li Y, Cao Y, Dela Mater R, et al. (2011) The effect of tobacco outlet density and proximity on smoking cessation. Am J Public Health 101(2): 315–320.
7. Halonen JI, Kivimaki M, Kouvonen A, Pentti J, Kawachi I, et al. (2013) Proximity to a tobacco store and smoking cessation: a cohort study. Tob Control. doi:10.1136/tobaccocontrol-2012-050726.
8. Henriksen L, Flora JA, Feighery EC, Fortmann SP (2002) Effects on youth of exposure to retail tobacco advertising. J Appl Soc Psychol 32(9): 1771–1789.
9. Henriksen L, Feighery EC, Wang Y, Fortmann SP (2004) Association of retail tobacco marketing with adolescent smoking. Am J Public Health 94(12): 2081–2083.
10. Lovato CY, Hsu HC, Sabiston CM, Hadd V, Nykiforuk CI (2007) Tobacco point-of-purchase marketing in school neighbourhoods and school smoking prevalence: a descriptive study. Can J Public Health 98(4): 265–270.
11. Slater SJ, Chaloupka FJ, Wakefield M, Johnston LD, O'Malley PM (2007) The impact of retail cigarette marketing practices on youth smoking uptake. Arch Pediatr Adolesc Med 161(5): 440–445.
12. Wakefield M, Germain D, Henriksen L (2008) The effect of retail cigarette pack displays on impulse purchase. Addiction 103(2): 322–328.
13. Burton S, Clark L, Jackson K (2012) The association between seeing retail displays of tobacco and tobacco smoking and purchase: findings from a diary-style survey. Addiction 107(1): 169–175.
14. Chapman S, Freeman B (2009) Regulating the tobacco retail environment: beyond reducing sales to minors. Tob Control 18(6): 496–501.
15. Yu D, Peterson NA, Sheffer MA, Reid RJ, Schnieder JE (2010) Tobacco outlet density and demographics: Analysing the relationships with a spatial regression approach. Public Health 124(7): 412–416.
16. Hyland A, Travers MJ, Cummings KM, Bauer J, Alford T, et al. (2003) Tobacco outlet density and demographics in Erie County, New York. Am J Public Health 93(7): 1075–1076.
17. Schneider JE, Reid RJ, Peterson NA, Lowe JB, Hughey J (2005) Tobacco outlet density and demographics at the tract level of analysis in Iowa: implications for environmentally based prevention initiatives. Prev Sci 6(4): 319–325.
18. Loomis BR, Kim AE, Goetz JL, Juster HR (2013) Density of tobacco retailers and its association with sociodemographic characteristics of communities across New York. Public Health 127(4): 333–338.
19. Rodriguez D, Carlos HA, Adachi-Mejia AM, Berke EM, Sargent JD (2013) Predictors of tobacco outlet density nationwide: a geographic analysis. Tob Control 22(5): 349–355.
20. Marsh L, Doscher C, Robertson LA (2013) Characteristics of tobacco retailers in New Zealand. Health Place 23: 165–170.
21. Reid RJ, Morton CM, Garcia-Reid P, Peterson NA, Yu D (2013) Examining tobacco outlet concentration in New Jersey: does income and ethnicity matter? J Ethn Subst Abuse 12(3): 197–209.
22. Schmitt CL, Juster HR, Dench D, Willett J, Curry LE (2013) Public and policy maker support for point-of-sale tobacco policies in New York. Am J Health Promot. doi:10.4278/ajhp.121023-QUAN-514.
23. Kroon LA, Corelli RL, Roth AP, Hudmon KS (2013) Public perceptions of the ban on tobacco sales in San Francisco pharmacies. Tob Control 22(6): 369–371.
24. Patwardhan P, McMillen R, Winickoff JP (2013) Consumer perceptions of the sale of tobacco products in pharmacies and grocery stores among U.S. adults. BMC Res Notes 6: 261.
25. Wiles T (2013) Youth group drafts proposal to limit tobacco retail. Available: http://missionlocal.org/2013/03/youth-group-drafts-proposal-to-limit-tobacco-retail/. Accessed 24 July 2013.
26. Wicked Local Brookline (2013) Buy some smokes? Minimum age is now 19 in Brookline. Available: http://www.wickedlocal.com/brookline/news/x1716634706/Buy-some-smokes-Minimum-age-is-now-19-in-Brookline. Accessed 24 July 2013.
27. Hartocollis A (2013) New York raising age to buy cigarettes to 21. New York Times. A21.
28. O'Connell J (2013) Ashland health board to discuss new tobacco regulations. Metrowest Daily News. Available: http://www.metrowestdailynews.com/news/x853687702/Ashland-health-board-to-discuss-new-tobacco-regulations. Accessed 24 July 2013.
29. Americans for Nonsmokers' Rights (2013) Tobacco-free pharmacies. Available: http://www.no-smoke.org/learnmore.php?id = 615. Accessed 13 August 2013.
30. Seidenberg AB, Hong W, Liu J, Noel JK, Rees VW (2013) Availability and range of tobacco products for sale in Massachusetts pharmacies. Tob Control 22(6): 372–375.
31. Hoek J, Vaudrey R, Gendall P, Edwards R, Thomson G (2012) Tobacco retail displays: a comparison of industry arguments and retailers' experiences. Tob Control 21(5): 497–501.
32. McDaniel PA, Malone RE (2011) Why California retailers stop selling tobacco products, and what their customers and employees think about it when they do: case studies. BMC Public Health 11: 848.
33. Fichtenberg CM, Glantz SA (2000) Association of the California Tobacco Control Program with declines in cigarette consumption and mortality from heart disease. N Engl J Med 343(24): 1772–1777.
34. QSR International (2010) NVivo qualitative data analysis software. 10 ed.
35. Hsieh HF, Shannon SE (2005) Three approaches to qualitative content analysis. Qual Health Res 15(9): 1277–1288.
36. McDaniel PA, Offen N, Yerger VB, Malone RE (2013) ''A breath of fresh air worth spreading'': Media coverage of retailer abandonment of tobacco sales. Am J Public Health. In press.
37. Patton MQ (2002) Qualitative research and evaluation methods. Thousand Oaks, CA: Sage Publications.
38. Federation of Tax Administrators (2013) Cigarette tax increases 2000–2013. Available: http://www.taxadmin.org/fta/rate/cig_inc02.html. Accessed 13 August 2013.

39. Campaign for Tobacco-Free Kids (2013) Top combined state-local cigarette tax rates. Available: http://www.tobaccofreekids.org/research/factsheets/pdf/0267.pdf. Accessed 13 August 2013.
40. McCormick R (2009) Cigarette vendors hit with huge increase in state licensing fee. Available: http://www.silive.com/news/index.ssf/2009/09/cigarette_vendors_hit_with_hug.html. Accessed 13 August 2013.
41. The New York State Department of Taxation and Finance (2012) Cigarette and tobacco products tax. Available: http://www.tax.ny.gov/bus/cig/cigidx.htm. Accessed 13 August 2013.
42. Center for Public Health and Tobacco Policy (2011) Tobacco retail licensing fees. Available: http://www.tobaccopolicycenter.org/program-areas/topic-areas/recent-cases/. Accessed 13 August 2013.
43. Summit County Fiscal Office (2013) Cigarette licensing. Available: https://fiscaloffice.summitoh.net/index.php/cigarette-licensing. Accessed 13 August 2013.
44. New York State Liquor Authority Division of Alcoholic Beverage Control (2013) Handbook for retail licenses. Available: http://www.sla.ny.gov/handbook-for-retail-licenses. Accessed 13 August 2013.
45. Lawriter Ohio Laws and Rules (2013) Chapter 4303 Liquor permits. Available: http://codes.ohio.gov/orc/4303. Accessed 13 August 2013.
46. O'Connell ME (2008) Decision deserves praise. The Allentown Morning Call. A8.
47. Anonymous (2008) Cigarette sales curtailed–Supermarket deserves credit for ban, but state mandate proposal goes too far. The Buffalo News. A6. Available: http://www.highbeam.com/doc/1P2-21666312.html. Accessed 27 December 2013.
48. Anonymous (2008) Editorial: Education best way to snuff smoking. Utica Observer-Dispatch.
49. Rivers T (2008) State only encourages scofflaw smoking with its sky-high tax. The Batavia Daily News. 4A.
50. Eshelman N (2008) Store blows smoke on cigarette sales. The Harrisburg Patriot-News.
51. Chaloupka FJ, Yurekli A, Fong GT (2012) Tobacco taxes as a tobacco control strategy. Tob Control 21(2): 172–180.
52. California Department of Public Health (2012) Healthy retailers, healthy communities: Integrating alcohol, tobacco, and healthy foods strategies. Available: http://www.cce.csus.edu/conferences/cdph/hrhc12/hrhc12.cfm?pid=13. Accessed 30 September 2013.
53. Eule B, Sullivan MK, Schroeder SA, Hudmon KS (2004) Merchandising of cigarettes in San Francisco pharmacies: 27 years later. Tob Control 13(4): 429–432.
54. Menashe CL, Siegel M (1998) The power of a frame: an analysis of newspaper coverage of tobacco issues–United States, 1985–1996. J Health Commun 3(4): 307–325.
55. Jacobson PD, Wasserman J, Raube K (1993) The politics of antismoking legislation. J Health Polit Policy Law 18(4): 787–819.
56. Daynard RA (2009) Doing the unthinkable (and saving millions of lives). Tob Control 18(1): 2–3.
57. Proctor RN (2013) Why ban the sale of cigarettes? The case for abolition. Tob Control (Suppl. 1): i27–i30.
58. Berrick AJ (2013) The tobacco-free generation proposal. Tob Control (Suppl. 1): i22–i26.
59. Khoo D, Chiam Y, Ng P, Berrick AJ, Koong HN (2010) Phasing-out tobacco: proposal to deny access to tobacco for those born from 2000. Tob Control 19(5): 355–360.
60. Malone RE (2013) Tobacco endgames: What they are and are not, issues for tobacco control strategic planning, and a possible US scenario. Tob Control (Suppl. 1): i42–i44.
